# Mre11-Rad50 Promotes Rapid Repair of DNA Damage in the Polyploid Archaeon *Haloferax volcanii* by Restraining Homologous Recombination

**DOI:** 10.1371/journal.pgen.1000552

**Published:** 2009-07-10

**Authors:** Stéphane Delmas, Lee Shunburne, Hien-Ping Ngo, Thorsten Allers

**Affiliations:** Institute of Genetics, School of Biology, University of Nottingham, Queen's Medical Centre, Nottingham, United Kingdom; Université Paris Descartes, INSERM U571, France

## Abstract

Polyploidy is frequent in nature and is a hallmark of cancer cells, but little is known about the strategy of DNA repair in polyploid organisms. We have studied DNA repair in the polyploid archaeon *Haloferax volcanii*, which contains up to 20 genome copies. We have focused on the role of Mre11 and Rad50 proteins, which are found in all domains of life and which form a complex that binds to and coordinates the repair of DNA double-strand breaks (DSBs). Surprisingly, *mre11 rad50* mutants are more resistant to DNA damage than the wild-type. However, wild-type cells recover faster from DNA damage, and pulsed-field gel electrophoresis shows that DNA double-strand breaks are repaired more slowly in *mre11 rad50* mutants. Using a plasmid repair assay, we show that wild-type and *mre11 rad50* cells use different strategies of DSB repair. In the wild-type, Mre11-Rad50 appears to prevent the repair of DSBs by homologous recombination (HR), allowing microhomology-mediated end-joining to act as the primary repair pathway. However, genetic analysis of recombination-defective *radA* mutants suggests that DNA repair in wild-type cells ultimately requires HR, therefore Mre11-Rad50 merely delays this mode of repair. In polyploid organisms, DSB repair by HR is potentially hazardous, since each DNA end will have multiple partners. We show that in the polyploid archaeon *H. volcanii* the repair of DSBs by HR is restrained by Mre11-Rad50. The unrestrained use of HR in *mre11 rad50* mutants enhances cell survival but leads to slow recovery from DNA damage, presumably due to difficulties in the resolution of DNA repair intermediates. Our results suggest that recombination might be similarly repressed in other polyploid organisms and at repetitive sequences in haploid and diploid species.

## Author Summary

Most organisms contain only one or two copies of their genome, but in some species multiple copies are found. The presence of multiple genome copies (polyploidy) has profound implications for DNA repair and is frequently seen in cancer cells. We have studied DNA repair in the archaeon *Haloferax volcanii*, which contains up to 20 genome copies. Archaea are a third form of life distinct from bacteria and eukaryotes. We have focused on the DNA repair proteins Mre11 and Rad50, which are found in virtually all organisms and which in humans act to prevent cancer. Surprisingly, we have found that *H. volcanii* cells deficient in Mre11-Rad50 are more resistant to DNA damage than wild-type cells. The DNA damage resistance of *mre11 rad50* mutant cells appears to be due to the exclusive use of homologous recombination, a DNA repair mechanism that is accurate but has the potential to generate genome rearrangements that require time to resolve. Correspondingly, we have found repair of DNA damage in *mre11 rad50* mutants takes longer than in wild-type cells. Our results suggest that polyploid organisms employ a program of DNA repair that minimizes their reliance on homologous recombination.

## Introduction

Bacterial and eukaryotic cells are normally assumed to be haploid and diploid, respectively, but polyploidy is surprisingly widespread. Polyploid cells can arise naturally during development of otherwise haploid or diploid organisms (e.g. hepatocytes), or as a consequence of cellular stress and disease (e.g. cancer, reviewed in [1]). Organisms that are constitutively polyploid are common amongst eukaryotes, and include plants, fish and amphibians. Polyploid bacteria include the radiotolerant species *Deinococcus radiodurans*, which harbors ∼8 copies of its genome [2], and *Epulopiscium* spp., which contain tens of thousands of genome copies [3]. Amongst archaea, *Methanocaldococcus jannaschii*, *Halobacterium salinarum* and *Haloferax volcanii* have been shown to be naturally polyploid [4],[5].

The presence of multiple genome copies affects many aspects of cell metabolism, in particular pathways of DNA repair. Since homologous recombination (HR) requires an identical genome copy, its usage for DNA repair is influenced by cell ploidy. When only one genome copy is present in the G1 phase of the eukaryotic cell cycle, DNA double-strand breaks (DSBs) are repaired by non-homologous end-joining (NHEJ), while HR is the predominant form of DSB repair in the G2 phase (reviewed in [6]). A further doubling of the ploidy of eukaryotic cells can result in increased reliance on HR, since genes involved in HR become essential for viability in tetraploid yeast [7]. In the presence of 8 genome copies in *D. radiodurans*, RecA-dependent HR is also required for DSB repair. However, HR is the second part of a two-stage DSB repair process, and is preceded by RecA-independent extended synthesis-dependent strand annealing [8].

It is a common assumption that additional genome copies might help protect polyploid cells from DNA damage. This is not the case, since tetraploid *Saccharomyces cerevisiae* cells are no more resistant to DNA damage than diploids [9],[10]. Furthermore, *D. radiodurans* cells have the same survival rate after ionizing radiation, whether they contain 4 or 10 genome copies [11].

We have undertaken a study of DNA repair in the halophilic archaeon *H. volcanii*, which is naturally polyploid and contains 10–20 copies of the genome, depending on growth phase [4]. Archaea are of great interest in their own right, and share many core components of their DNA processing machinery with eukaryotes (reviewed in [12]). We have focused on the role of the Mre11-Rad50 complex, which is present in all domains of life and is involved in several pathways of DSB repair including HR and NHEJ (reviewed in [13]). Mre11 is a nuclease, while Rad50 consists of two globular DNA-binding domains (reviewed in [14]). Together, Mre11 and Rad50 form a complex that binds to and tethers DNA ends, in order to erect a scaffold for the subsequent processing and repair of DSBs [15]–[17]. For instance, Mre11-Rad50 has recently been shown to initiate 5′-strand resection at DSBs [18],[19].

Mre11-Rad50 is critical for DSB repair, and *S. cerevisiae* mutants in *mre11* or *rad50* are acutely sensitive to agents that induce DSBs [20],[21]. Mre11 is one of the first proteins to localize to the sites of DSBs [22], where it activates the ATM/Tel1 kinase that is central to the DNA damage-induced checkpoint [23]. It is noteworthy that Mre11 foci at the sites of DSBs dissociate before the appearance of “classical” HR proteins such as Rad51 and Rad52 [22]. The temporal separation between binding by Mre11-Rad50 and the subsequent repair of DSBs presumably allows for the appropriate pathway (HR or NHEJ) to be chosen. Mre11-Rad50 is also essential for the repair of meiotic DSBs in both *S. cerevisiae* and *Schizosaccharomyces pombe*, but only in *S. cerevisiae* does the formation of meiotic DSBs depend on Mre11-Rad50 [24],[25]. Similar differences are found with respect to NHEJ, where Mre11-Rad50 is required for NHEJ in *S. cerevisiae* but not in *S. pombe* [26]–[28].

The bacterial homolog of Mre11-Rad50 is SbcCD. Sensitivity to DNA damage is also seen in *sbcCD* mutants of some bacterial species such as *D. radiodurans* and *Bacillus subtilis* [29],[30], and *D. radiodurans sbcCD* cells exhibit delayed repair of DSBs after ionizing radiation [29]. Similarly retarded kinetics of DSB repair is seen in *mre11* mutants of the archaeon *Halobacterium* sp. NRC-1, although in this species deletion of *mre11* or *rad50* does not result in sensitivity to DNA damage [31]. We have deleted *mre11* and *rad50* genes in the polyploid archaeon *H. volcanii* and have found that mutants are more resistant to DNA damage than the wild-type. Our results indicate that the use of HR is restrained by Mre11-Rad50, and that the unrestrained use of HR in *mre11 rad50* mutants enhances cell survival but leads to slower recovery from DNA damage.

## Results

**Figure 1:**
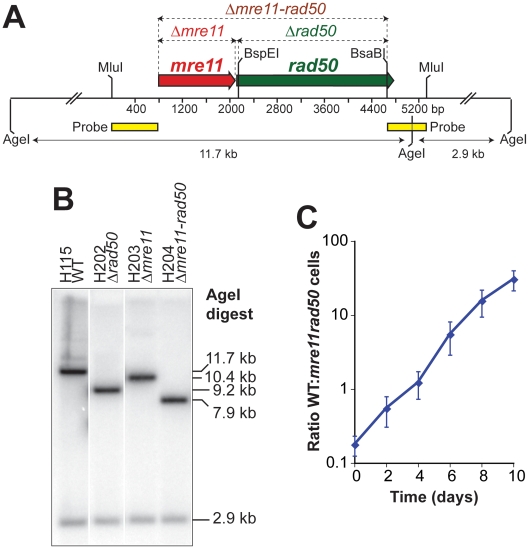
Construction of *mre11 rad50* mutants. (A) Map of *mre11-rad50* operon of *H. volcanii* indicating MluI sites used to isolate the genome clone, location of the *mre11* and *rad50* deletions, and AgeI sites and probe used to verify the deletions. (B) Southern blot of genomic DNA cut with AgeI, probed with DNA flanking *mre11-rad50* operon, to indicate deletion of *mre11* and/or *rad50* genes. (C) *mre11 rad50* mutants show a growth defect of ∼1% per generation. WT and *mre11 rad50* strains (H115 and H204, respectively) were mixed in a ∼1∶10 ratio, respectively, and grown together in pairwise competition. The average and standard error (SE) of four experiments are shown.

The *mre11* and *rad50* genes were identified in an operon in the *H. volcanii* genome (Figure 1A). All motifs diagnostic for Mre11 and Rad50 [32] are conserved in the *H. volcanii* proteins (Figure S1). Genes for NurA and HerA, which cluster with *mre11* and *rad50* in thermophilic archaea [33],[34], are not apparent in the *H. volcanii* sequence. Xrs2 and Nbs1, which form part of the Mre11-Rad50 complex in yeast and higher eukaryotes respectively [13],[14], are not found in archaea. Sequence analysis of the *mre11-rad50* region failed to identify additional genes in the operon.

Deletion mutants of *rad50*, *mre11*, and *mre11 rad50* were constructed using a gene knockout system for *H. volcanii* (Figure 1B) [35]. The generation time of the mutants during exponential growth in rich medium (Hv-YPC broth) was similar to the wild-type (WT) (∼2 hours). However, in a pairwise growth competition assay, the *mre11 rad50* mutant was out-competed by the WT (Figure 1C). The growth advantage of the WT is ∼1% per generation.

### *mre11 rad50* Mutants Show Enhanced Resistance to DNA Damaging Agents, but Recover More Slowly Than the Wild-Type

**Figure 2:**
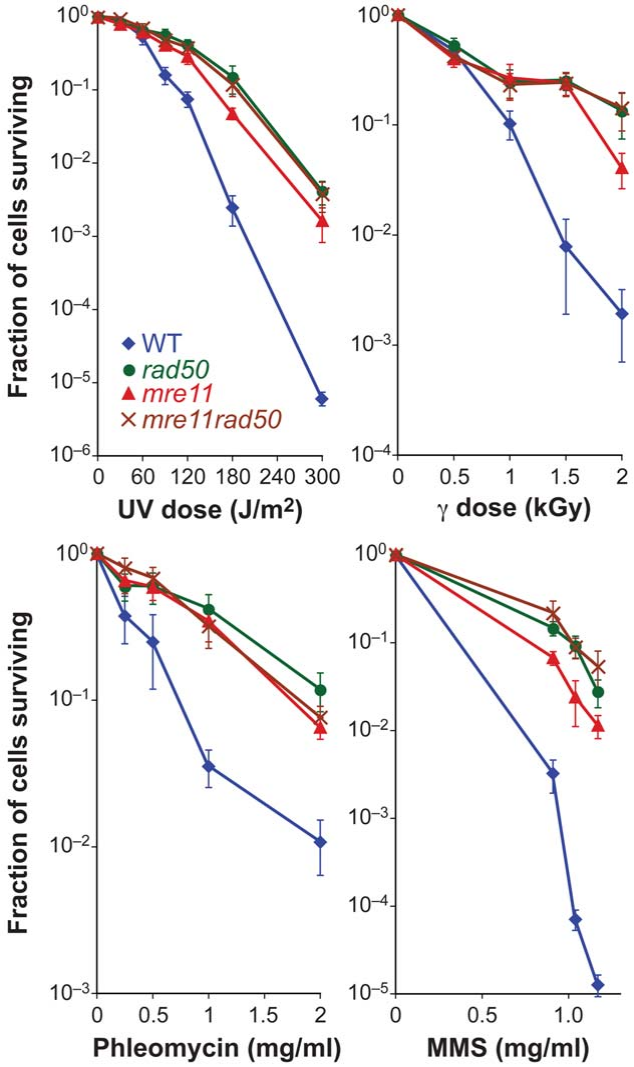
*mre11 rad50* mutants show increased resistance to DNA damage. WT, *rad50*, *mre11* and *mre11 rad50* cultures (H115, H202, H203, and H204, respectively) were plated and exposed to ultraviolet (UV) or γ radiation. Alternatively, phleomycin or methyl methanesulphonate (MMS) was added and cultures were incubated at 45°C for 1 hour before plating. In each case, the average and SE of six experiments are shown.

We examined the sensitivity of *H. volcanii rad50*, *mre11* and *mre11 rad50* mutants to DNA damage, specifically ultraviolet (UV) and γ radiation, the radiomimetic chemical phleomycin, and the alkylating agent methyl methanesulphonate (MMS). In all cases, the mutants are significantly more resistant to DNA damage than the WT strain (Figure 2). The UV sensitivity of *mre11 rad50* mutants was restored to WT levels by expression of the *mre11-rad50* operon from a replicative plasmid (pTA795), confirming that hyper-resistance to DNA damage is due to *mre11 rad50* deletion (data not shown).

**Figure 3:**
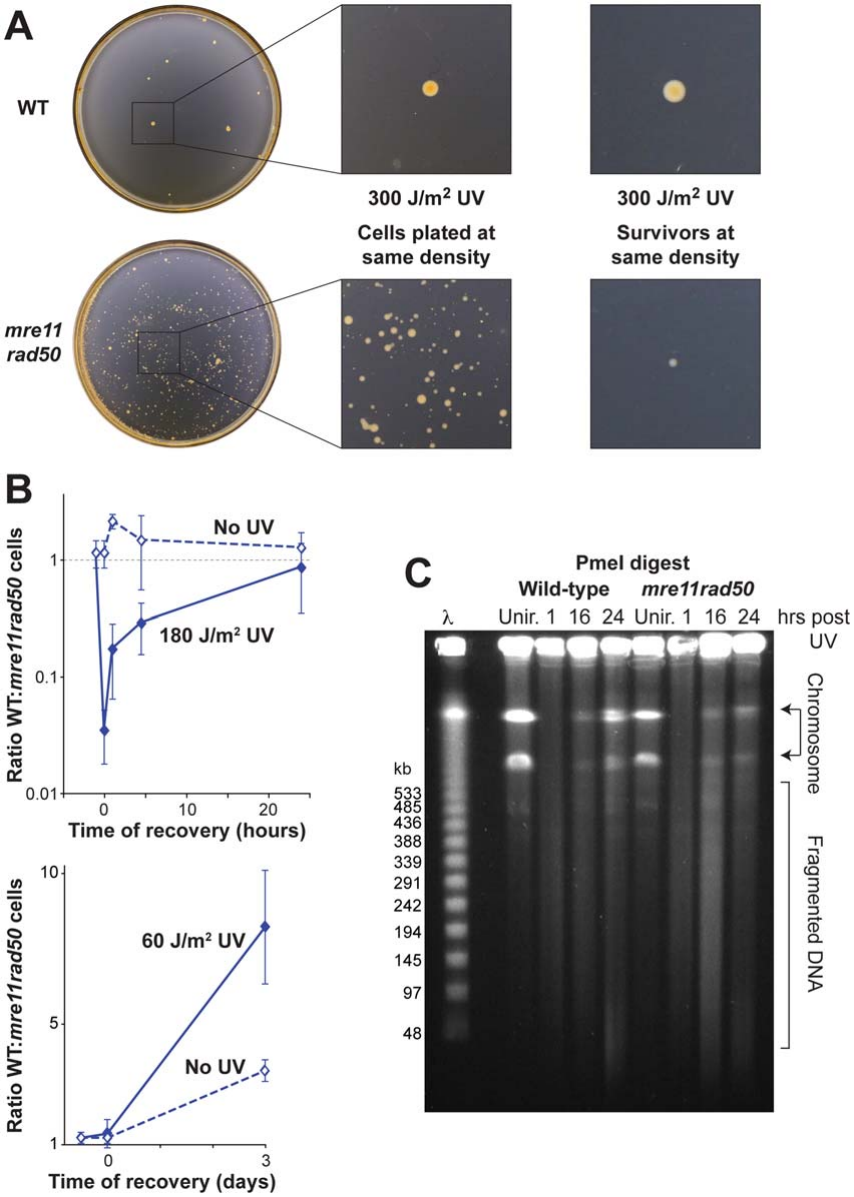
WT cells recover faster from UV irradiation than *mre11 rad50* mutants. (A) *mre11 rad50* UV survivors show retarded colony growth. WT and *mre11 rad50* cultures (H115 and H204, respectively) were plated at the same density, exposed to 300 J/m^2^ UV, and grown for 5 days. Similar results were seen with *mre11* and *rad50* single mutants (data not shown). Photographs on the right show colony sizes formed after UV irradiation, where cells were plated at differing densities to ensure equal survival (∼10 colonies/plate), thus avoiding effects of poorer growth due to crowding. (B) WT survivors recover from UV irradiation faster than *mre11 rad50* mutants. WT and *mre11 rad50* strains (H115/H642 and H204/H645, respectively) were mixed in a 1∶1 ratio, irradiated with 180 J/m^2^ or 60 J/m^2^ UV, and grown in pairwise competition. Aliquots were plated at regular intervals and the fraction of WT cells determined. The average and SE of ≥six experiments are shown. (C) *mre11 rad50* mutants exhibit delayed repair of DNA damage. Samples were taken immediately before (unirradiated, Unir.) and at 1, 16 and 24 hours after irradiation with 180 J/m^2^ UV. Genomic DNA in agarose plugs was digested with PmeI (which cuts the main chromosome twice) and subject to pulsed-field gel electrophoresis. Representative image taken from one of four independent replicates. Similar results were seen with *mre11* and *rad50* single mutants (data not shown).

After UV irradiation, *mre11 rad50* colonies were smaller than WT colonies (Figure 3A). The small *mre11 rad50* colonies yielded normal-sized colonies on restreaking (data not shown), therefore the small-colony phenotype is probably due to a temporary delay in growth of *mre11 rad50* cells after UV irradiation. To investigate this further, we carried out pairwise growth competition assays after UV irradiation (Figure 3B). After 180 J/m^2^ UV, only a small fraction of WT cells survive, but these survivors exhibit a rapid recovery that results in restoration of the WT cell fraction to pre-UV levels after 24 hours. Irradiation with 60 J/m^2^ UV results in <50% cell death and there is no difference in survival between WT and mutant cells (Figure 2), but pairwise growth competition shows that the WT has a significantly faster recovery from DNA damage than the *mre11 rad50* mutant (Figure 3B).

Repair of DNA damage was monitored directly by pulsed-field gel electrophoresis. Within 1 hour after irradiation with 180 J/m^2^ UV, the genome is fragmented by DSBs (Figure 3C). Formation of these DSBs requires processing of UV-induced DNA damage, since the total disappearance of bands corresponding to an intact chromosome is not seen until 10–30 minutes after irradiation (data not shown). Bands corresponding to an intact chromosome are visible again by 16 hours in both WT and *mre11 rad50* cells, but in contrast to the WT, the majority of DNA in the *mre11 rad50* mutant is found in a characteristic smear of broken fragments. By 24 hours, the WT has reconstituted the genome, whereas fragmented DNA still persists in the *mre11 rad50* mutant. Therefore, repair of DNA damage is more rapid in the WT than the mutant.

### Homologous Recombination Is the Primary Pathway of DSB Repair in *mre11 rad50* Mutants, but Not in the Wild-Type

**Figure 4:**
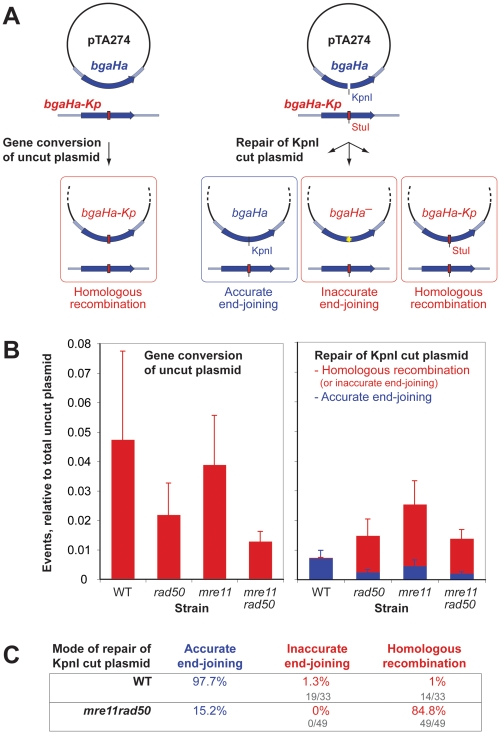
Compared to wild-type, *mre11 rad50* mutants show elevated levels of homologous recombination at DSBs. (A) Plasmid repair assay. pTA274 carries a 3 kb genomic fragment (light blue) with the *bgaHa* beta-galactosidase gene (dark blue). The chromosomal *bgaHa-Kp* allele contains a 26 bp oligonucleotide (with a novel StuI site) at the KpnI site. Uncut plasmid may be gene converted by the chromosomal *bgaHa-Kp* allele, while plasmid cut at the KpnI site may be repaired in three ways: (1) accurate joining of KpnI-cut ends to restore *bgaHa*, resulting in blue colonies when stained with Xgal; (2) inaccurate joining of the DSB; (3) HR with the chromosomal *bgaHa-Kp* allele. The latter two outcomes result in red colonies that do not stain with Xgal, and are distinguished by a StuI digest of plasmid DNA. (B) WT and *mre11 rad50* cell repair DSBs differently. WT, *rad50*, *mre11* and *mre11 rad50* strains (H115, H202, H203, and H204, respectively) were transformed with either uncut (left graph) or KpnI-cut pTA274 (right graph). The left graph shows gene conversion of uncut plasmid. The right graph shows repair of KpnI-cut plasmid, relative to transformation efficiency with uncut plasmid (total transformants); blue bars represent accurate end-joining, and red bars represent HR or inaccurate end-joining. The average and SE of 5 independent transformations are shown. (C) Repair in WT cells is primarily by end-joining, while *mre11 rad50* mutants use mainly HR. All red *mre11 rad50* transformants examined (n = 49) contained a plasmid with a StuI site (HR), whereas half the red WT transformants examined (n = 33) contained a plasmid that failed to cut with StuI (inaccurate end-joining).

We examined DSB repair using the recombination assay shown in Figure 4A. Cells are transformed with a replicative plasmid carrying the beta-galactosidase gene *bgaHa* (Text S1, Figure S3). This allele can recombine with a mutant *bgaHa-Kp* allele on the chromosome. If the plasmid is cut with KpnI prior to transformation, the DSB can be repaired either by end-joining or by HR. Repair by accurate end-joining (religation) results in colonies that stain blue with Xgal, whereas inaccurate end-joining or HR results in colonies that remain red (*bgaHa^−^*). Inaccurate end-joining and HR can be distinguished by a restriction digest of plasmid DNA, since the StuI site in the *bgaHa-Kp* allele is diagnostic of HR (Figure 4A and 4C).

If the plasmid is left uncut, gene conversion of the plasmid-borne *bgaHa* allele does not differ significantly between the WT and the *mre11 rad50* mutants (Figure 4B, left graph). Therefore, inactivation of Mre11-Rad50 does not affect HR of circular DNA. If the plasmid is cut with KpnI, the efficiency of DSB repair is similar in the WT and mutants, however the mode of DSB repair differs markedly between these strains (Figure 4B, right graph). In the WT, the vast majority of DSBs are repaired by accurate end-joining, with very little contribution of HR or inaccurate end-joining (Figure 4B and 4C). By contrast, in *mre11 rad50* mutants most repair is by HR, while accurate end-joining is reduced by ∼50%. It is notable that in the WT, the frequency of HR between the plasmid and chromosome is reduced almost 300-fold when the plasmid is cut with KpnI, whereas in the *mre11 rad50* mutants HR is not affected by the presence of a DSB (Figure 4B, compare left and right graphs). Therefore, Mre11-Rad50 restrains the use of HR at DSBs.

Since Mre11 is a nuclease and Mre11-Rad50 has been shown to initiate 5′-strand resection at DSBs [18],[19],[36], it is possible that *H. volcanii* Mre11-Rad50 might prevent HR by nucleolytic degradation of DSBs. To test this, we modified the DSB assay by inserting a *trpA* marker at the end of the plasmid-borne *bgaHa* gene (Figure S2). Degradation of KpnI-cut DNA is measured by loss of the *trpA* marker, but HR with the chromosomal *bgaHa-Kp* allele is still possible using downstream sequences. There was no significant difference between WT and mutant strains in the frequency of marker loss, suggesting that Mre11 is not responsible for DSB degradation in *H. volcanii*.

We also measured the fraction of single-stranded DNA in WT and *mre11 rad50* cells, by slot-blotting of native (undenatured) and denatured genomic DNA, and hybridization with a genomic DNA probe (Figure S2C). After irradiation with 180 J/m^2^ UV, the fraction of single-stranded DNA increases ∼2.5-fold (Figure S2D), but there was no significant difference between WT and *mre11 rad50* strains. Thus, Mre11-Rad50 is not responsible for the formation of single-stranded DNA after UV damage.

Mre11 has been shown to facilitate end-joining at microhomologies *in vitro* [37]. In WT *H. volcanii*, repair of the cut plasmid is primarily by accurate re-ligation of cohesive KpnI ends. The efficiency of this end-joining is reduced by ∼50% in *mre11 rad50* mutants, suggesting that it is partially dependent on Mre11-Rad50. End-joining also depends on micro-homology, since its efficiency is decreased by >90% if the DSB is blunt-ended (data not shown). Furthermore, plasmid repair events by inaccurate end-joining are characterized by deletions of the *bgaHa* gene, which are flanked by microhomologies of 3–5 bp (Figure S2E). This repair pathway is Ku-independent, since *H. volcanii* (and almost all other archaea) does not encode a Ku homolog [38], and resembles the microhomology-mediated end-joining seen in other organisms [39].

### DNA Repair in Both Wild-Type and *mre11 rad50* Cells Requires RadA Recombinase

**Figure 5:**
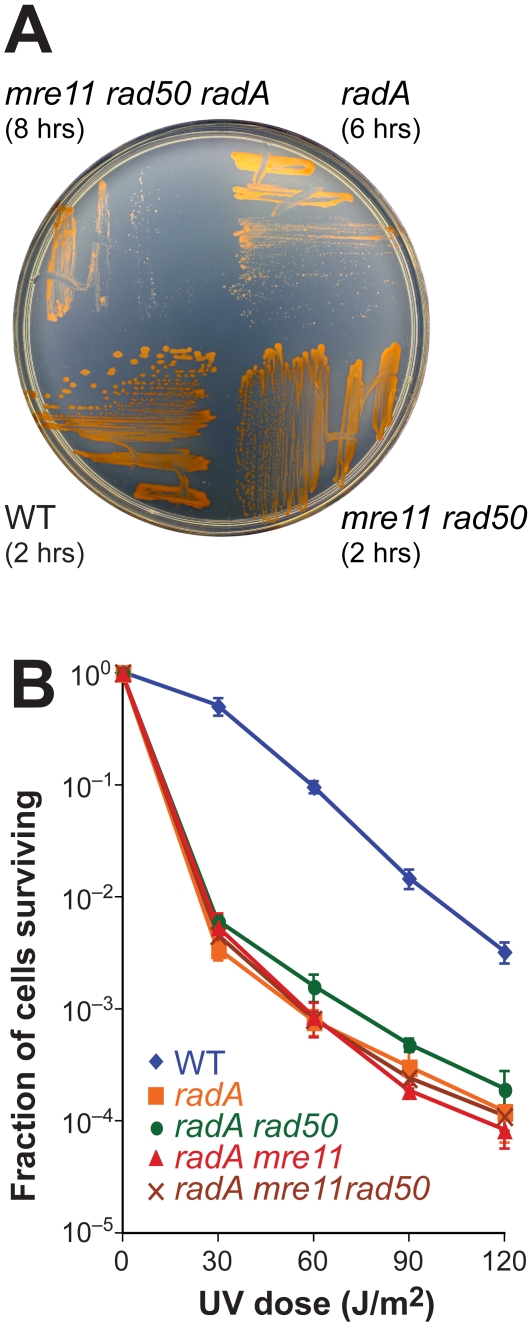
Repair of UV–induced DNA damage ultimately depends on RadA. (A) Deletion of *mre11 rad50* and *radA* leads to a growth defect that is worse than *radA* alone. WT, *mre11 rad50*, *radA* and *mre11 rad50 radA* strains (H195, H280, H387, and H392, respectively) were grown for 10 days on Hv-YPC+Thy plates. The generation times of these strains in Hv-YPC+Thy broth are indicated in parentheses. The phenotypes of *mre11 radA* (H448) and *rad50 radA* (H389) mutants are similar to that of the *mre11 rad50 radA* strain (data not shown). (B) *mre11 rad50* deletion does not enhance the UV-sensitivity of a *radA* mutant. WT, *radA*, *rad50 radA*, *mre11 radA*, and *mre11 rad50 radA* cultures (H195, H387, H389, H448, and H392, respectively) were plated and exposed to UV. The average and SE of six experiments are shown.

RadA is the *H. volcanii* ortholog of RecA/Rad51 recombinase and is more similar to eukaryotic Rad51 than bacterial RecA [40]. Mutants of *radA* are slow-growing, sensitive to DNA damage, and completely deficient in recombination [41]. However, microhomology-mediated end-joining of a cut plasmid (as described in Figure 4) is observed in a *radA* mutant (H607, data not shown). Deletion of *radA* in an *mre11 rad50* background leads to a growth defect that is worse than that seen with *radA* alone (Figure 5A). This might suggest that Mre11-Rad50 and RadA act in different pathways of DNA repair. However, we found that *radA mre11 rad50* mutants are no more sensitive to UV radiation than *radA* strains (Figure 5B). This indicates that in both WT and *mre11 rad50* cells, the repair of UV-induced DNA damage requires RadA, and therefore most likely HR.

## Discussion

We have deleted the *mre11* and *rad50* genes of the polyploid archaeon *H. volcanii* and have found that: (1) *mre11 rad50* mutants are hyper-resistant to DNA damage, but recover from DNA damage and repair DSBs more slowly than the WT; (2) *mre11 rad50* mutants exhibit more homologous recombination at DSBs than the WT; (3) RadA recombinase is ultimately required for DNA repair.

### Absence of Mre11-Rad50 Increases Resistance to DNA Damage

In *S. cerevisiae*, null mutations of *mre11* or *rad50* confer sensitivity to ionizing radiation and MMS, although not to UV [20],[21]. In bacteria, *Deinococcus radiodurans* and *Bacillus subtilis sbcCD* mutants are sensitive to DNA damage [29],[30]. However, it is not always the case that lack of Mre11-Rad50 or SbcCD results in decreased resistance to DNA damage. *E. coli sbcC* mutants and *mre11 rad50* mutants of the archaeon *Halobacterium* sp. NRC-1 do not show increased sensitivity to DNA damage [31],[42]. Intriguingly, we have found that *H. volcanii mre11* and *rad50* mutants are more resistant to DNA damage than the WT (Figure 2).

Mutations in DNA repair genes that increase resistance to DNA damage have been reported elsewhere. Defects in DNA ligase IV or the end-binding Ku70-Ku80 complex increase the resistance of chicken DT40 and yeast cells to high doses of γ radiation or phleomycin [43],[44]. The increased DNA damage resistance of NHEJ-defective cells is thought to be due to a failure to suppress HR [45]. Our work shows that this is also the case for *mre11 rad50* mutants of *H. volcanii*, since the hyper-resistance to DNA damage correlates with increased repair by HR.

### Mre11-Rad50 Promotes Rapid Recovery from DNA Damage

Although *mre11 rad50* mutants of *H. volcanii* are more resistant to DNA damage than the WT, they take longer to recover. This is evident in the size of colonies formed by surviving cells (Figure 3A), and in pairwise competition assays after UV irradiation (Figure 3B, left graph). WT survivors are less numerous but recover more rapidly than *mre11 rad50* mutants after irradiation with high doses of UV. This suggests that WT and *mre11 rad50* cells use two different DNA repair strategies. The speed of the Mre11-Rad50-dependent repair strategy gives WT cells a long-term advantage, in spite of being incapable of repairing high levels of DNA damage. At lower doses of DNA damage (60 J/m^2^ UV), the advantage of the WT repair strategy is also apparent (Figure 3B, right graph). One reason might be slower repair of DSBs in *mre11 rad50* mutants, as indicated by pulsed-field gels of genomic DNA after UV irradiation (Figure 3C). A similar delay in the repair of DSBs has been observed in *sbcCD* mutants of *D. radiodurans* and *mre11* mutants of the archaeon *Halobacterium* sp. NRC-1 [29],[31].

### Mre11-Rad50 Reduces Dependence on Homologous Recombination to Repair DSBs

Our results indicate that the DNA repair strategy used in *mre11 rad50* cells involves unrestrained HR. In WT cells, cut plasmid molecules are repaired by microhomology-mediated end-joining, whereas in the absence of Mre11-Rad50, HR is increased ∼100-fold and is the predominant mode of repair (Figure 4). This is in contrast to *S. cerevisiae*, where deletion of *mre11* reduces HR between plasmid and chromosome ∼20-fold [46]. Furthermore, in *S. cerevisiae* the presence of a DSB stimulates HR between plasmid and chromosome [47], whereas in WT *H. volcanii* the presence of a DSB reduces HR between plasmid and chromosome (Figure 4B, compare left and right graphs). This suggests that in *H. volcanii*, the preferred substrate for HR is not a DSB.

There are two (non-exclusive) hypotheses to account for our results: (1) Mre11-Rad50 binds to DSBs and directly prevents HR (e.g. by blocking assembly of RadA filaments); (2) Mre11-Rad50 stimulates an alternative pathway of DSB repair (e.g. by microhomology-mediated end-joining), thereby removing the substrate for HR. If the sole function of Mre11-Rad50 is to promote an alternative to HR, then mutations that eliminate HR should be synergistic with deletion of *mre11 rad50*. Mutation of *radA* renders cells sensitive to DNA damage and deficient in HR [41], and we show here that *radA mre11 rad50* mutants are no more sensitive to UV radiation than *radA* cells (Figure 5B). RadA might have other roles in addition to HR, such as activation of an SOS response to DNA damage, as seen in bacteria [48]. However, to date there is no evidence for an SOS response in archaea [49],[50]. For these reasons we favor the first hypothesis, where Mre11-Rad50 directly restrains HR and allows another pathway to act as the primary mode of DSB repair. The phenotype of *radA mre11 rad50* mutants suggests that HR is ultimately required for repair of DSBs, therefore the restraint on HR imposed by Mre11-Rad50 can only be temporary.

### Conclusions

Why does *H. volcanii* Mre11-Rad50 appear to act differently when compared to other organisms? We suggest that HR is restrained in *H. volcanii* because this species is highly polyploid [4]. Coordinating HR is likely to be problematic when each DSB has 20 partners to choose from. This problem is exacerbated in organisms with a circular genome, since DSB repair by HR runs the risk of generating chromosome concatemers, which require resolution before cell division. However, our results with *radA* mutants suggest that DNA repair ultimately requires HR. We propose that Mre11-Rad50 temporarily restrains HR, and promotes (directly or indirectly) a repair mechanism that reduces the number of DNA ends, which ultimately have to be repaired by HR. A two-step process of DNA repair has been proposed for the polyploid bacterium *D. radiodurans*, where DNA fragments are first reassembled by extended synthesis-dependent strand annealing (ESDSA) before chromosome reconstitution by HR [8]. However, the initial step used in *H. volcanii* is unlikely to be ESDSA, since we find no evidence for increased DNA synthesis after UV irradiation (data not shown). As we suggest above, *H. volcanii* might use microhomology-mediated end-joining, but other mechanisms for the initial DNA repair are also possible. In any case, this initial mechanism appears to be incapable of repairing large numbers of DSBs, as evident by the hyper-resistance to DNA damage seen in *mre11 rad50* mutants.

Mre11-Rad50 might also restrain HR in order to promote a physiological change, such as induction of a DNA damage response that allows time for processing of DSBs, and/or arrest at a cell cycle checkpoint. In eukaryotes, hyper-recombination is a common phenotype of checkpoint defective cells [51], and the small size of *H. volcanii mre11 rad50* colonies after UV irradiation (Figure 3A) is strikingly reminiscent of yeast cells that have adapted to the DNA damage checkpoint and re-entered the cell cycle [52].

Why would evolution restrain HR, if the unrestrained use of HR (in *H. volcanii mre11 rad50* mutants) results in higher cell survival? Yeast and chicken DT40 cells defective in NHEJ show increased DNA damage resistance, which is suggested to be due to a failure to suppress HR [43],[44]. Perhaps the cost of HR results from the time taken to repair DNA damage, and this cost is particularly acute for polyploid organisms. In the polyploid species *D. radiodurans* and *Halobacterium* sp. NRC-1, mutations in *sbcCD* and *mre11*, respectively, result in slower repair of DSBs [29],[31], similar to what we have observed for *H. volcanii*. We suggest that HR is restrained in these and other polyploid organisms, and at repetitive sequences in haploid/diploid species, for example the rDNA locus [53]. When many copies of a gene or genome are present, restraining HR might be necessary to prevent each DNA end from engaging with multiple partners.

## Materials and Methods

Unless stated otherwise, chemicals were from Sigma and restriction enzymes from New England Biolabs. Standard molecular techniques were used [54].

### Strains and Plasmids

*H. volcanii* strains are shown in Table 1, plasmids in Table S1, and oligonucleotides in Table S2. *H. volcanii* strains were grown at 45°C on complete (Hv-YPC) or casamino acids (Hv-Ca) agar, or in Hv-YPC or Hv-Ca broth, as described previously [55],[56]. To estimate generation times, a culture of Hv-YPC broth (+thymidine) was inoculated with ∼10^3^ cells/ml, viable cells/ml was determined by plating aliquots at regular intervals. Isolation of genomic and plasmid DNA, and transformation of *H. volcanii* were carried out as described previously [55],[57].

**Table 1:** *H. volcanii* strains.

Strain	Genotype	Derivation^a^
H26	*ΔpyrE2*	[55]
H54	*ΔpyrE2 bgaHa*	H26 pTA102
H115	*ΔpyrE2 bgaHa-Kp*	H54 pTA154
H202	*ΔpyrE2 bgaHa-Kp Δrad50*	H115 pTA137
H203	*ΔpyrE2 bgaHa-Kp Δmre11*	H115 pTA138
H204	*ΔpyrE2 bgaHa-Kp Δmre11 Δrad50*	H115 pTA171
H642	*ΔpyrE2 bgaHa*	H115 pTA151
H645	*ΔpyrE2 bgaHa Δmre11 Δrad50*	H204 pTA151
H292	*ΔpyrE2 bgaHa-Kp ΔtrpA*	H115 pTA95
H293	*ΔpyrE2 bgaHa-Kp ΔtrpA Δrad50*	H202 pTA95
H294	*ΔpyrE2 bgaHa-Kp ΔtrpA Δmre11*	H203 pTA95
H295	*ΔpyrE2 bgaHa-Kp ΔtrpA Δmre11 Δrad50*	H204 pTA95
H607	*ΔpyrE2 bgaHa-Kp ΔtrpA ΔradA::trpA^+^*	H292 pTA324
H195	*ΔpyrE2 bgaHa-Bb leuB-Ag1 ΔtrpA ΔhdrB*	[56]
H273	*ΔpyrE2 bgaHa-Bb leuB-Ag1 ΔtrpA ΔhdrB Δrad50*	H195 pTA137
H276	*ΔpyrE2 bgaHa-Bb leuB-Ag1 ΔtrpA ΔhdrB Δmre11*	H195 pTA138
H280	*ΔpyrE2 bgaHa-Bb leuB-Ag1 ΔtrpA ΔhdrB Δmre11 Δrad50*	H195 pTA171
H387	*ΔpyrE2 bgaHa-Bb leuB-Ag1 ΔtrpA ΔhdrB ΔradA::trpA^+^*	H195 pTA324^b^
H389	*ΔpyrE2 bgaHa-Bb leuB-Ag1 ΔtrpA ΔhdrB Δrad50 ΔradA::trpA^+^*	H273 pTA324^b^
H448	*ΔpyrE2 bgaHa-Bb leuB-Ag1 ΔtrpA ΔhdrB Δmre11 ΔradA::trpA^+^*	H276 pTA324^b^
H392	*ΔpyrE2 bgaHa-Bb leuB-Ag1 ΔtrpA ΔhdrB Δmre11 Δrad50 ΔradA::trpA^+^*	H280 pTA324^b^
H112	*ΔpyrE2 ΔradA*	[57]

a Parental strains and plasmids used in gene knockouts [35],[55]. Unless indicated otherwise, source of strains was this study.

b pTA411 also used (see Text S1).

### Cloning and Deletion of *mre11-rad50*

The *rad50* gene was identified in the genome sequence. A 469 bp fragment of *rad50* was amplified by PCR and used to probe a Southern blot of genomic DNA digested with MluI; a 5.3 kb fragment hybridized with the probe. A genomic library of MluI 5.3 kb fragments was constructed and screened by colony hybridization with the *rad50* probe. One clone (pTA42) was found to contain the *mre11-rad50* operon (Figure 1A). The sequence of the *mre11-rad50* operon has been deposited in the EMBL database (accession number AJ635369).

### Construction of *rad50*, *mre11*, and *mre11 rad50* Strains

To generate the *Δrad50* construct, a BspEI–BsaBI *rad50* fragment of pTA42 was deleted (Figure 1A). The *Δmre11* construct was generated by PCR, to ensure precise gene deletion that would not exert a polar effect on *rad50* (see Table S2). To generate the *Δmre11Δrad50* construct, the BspEI–BsaBI *rad50* fragment was deleted from the *Δmre11* construct. A *pyrE2* marker from pGB70 [35] was inserted into plasmids carrying *Δrad50*, *Δmre11* and *Δmre11 Δrad50* constructs, generating pTA137, pTA138 and pTA171 respectively, which were used to construct deletion strains by a gene knockout system [35]. Deletion of *radA* in *mre11 rad50* strains required complementation by a plasmid-borne *radA* gene (see Text S1 and Figure S4).

### Pairwise Growth Competition Assays

To compare strains during normal growth, a 10 ml culture of Hv-YPC broth was inoculated with ∼10^3^ WT and ∼10^4^ *mre11 rad50* cells (1∶10 ratio). The mixed culture was grown for 2 days (∼10^8^ cell/ml), diluted 1000-fold and ∼10^4^ cells was used to inoculate 10 ml Hv-YPC; this was repeated a further 3 times. After each inoculation, the ratio of WT and mutant cells was determined by plating, transferring 100 colonies to nylon membranes and probing with the 469 bp *rad50* PCR product. To compare the ratio of WT and mutant cells after UV irradiation, *bgaHa^+^* derivatives of H115 and H204 that stain blue with Xgal (5-bromo-4-chloro-3-indolyl-β-d-galactopyranoside) were generated (H642 and H645, respectively, Text S1 and Figure S3). Cultures were grown to ∼10^8^ cell/ml, centrifuged and resuspended in an equal volume of 18% SW (salt water) [55]. A 1∶1 mixture of WT and *mre11 rad50* cells (differentially marked with *bgaHa^+^* or *bgaHa-Kp*) was exposed to 180 J/m^2^ UV (254 nm, 1 J/m^2^/s) or left unirradiated. The mixed culture was centrifuged, resuspended in an equal volume of Hv-YPC broth and grown in the dark at 45°C. Aliquots of 10 µl were taken at the intervals shown and plated on Hv-YPC. After 5 days incubation, plates were sprayed with Xgal solution (BlueTech, Mirador) and cells scored the next day. Pairwise growth competition after 60 J/m^2^ UV was performed in a similar manner, except that after irradiation the culture was diluted 10^6^-fold in 1 ml Hv-YPC broth and grown in the dark for 3 days.

### DNA Damage Assays

For radiation assays, cultures were grown to ∼10^8^ cell/ml, diluted in 18% SW and 20 µl aliquots spotted on Hv-YPC plates. Once spots had dried, cells were exposed to UV or γ radiation (^137^Cs, 395 Gy/s). After UV exposure, cells were shielded from visible light. For chemical mutagen assays, cultures were divided into 1 ml aliquots, and phleomycin (Apollo Scientific) or methyl methanesulphonate was added. Cultures were returned to 45°C for 1 hour, diluted in 18% SW and plated on Hv-YPC. Survivors were counted after 5–14 days incubation.

### Pulsed Field Gel Electrophoresis

Cultures were grown to ∼10^8^ cell/ml, centrifuged, and resuspended in an equal volume of 18% SW. Cells were exposed to 180 J/m^2^ UV with shaking, centrifuged, resuspended in an equal volume of Hv-YPC broth and grown in the dark at 45°C. Samples were taken at the time indicated. Cells were centrifuged, resuspended in 20 µl 18% SW and embedded in 100 µl plugs at a final concentration of 0.8% SeaPlaque GTG agarose (Cambrex) prepared in 18% SW. Agarose plugs were incubated for ≥16 hours at 45°C in lysis solution (20 mM Tris-HCl pH 8.8, 500 mM EDTA pH 8, 1% N-lauroylsarcosine, 1 mg/ml proteinase K), then transferred to fresh lysis solution containing 10 µg/ml RNaseA for 4 hours at 45°C. Plugs were incubated in wash solution (25 mM Tris-HCl pH 7.5, 100 mM EDTA pH 8) for 30 minutes at 37°C, then transferred to fresh wash solution containing 1 mM phenylmethane sulfonyl fluoride for 1 hour at 37°C. Plugs were washed two more times for 30 minutes each, the final time in 1/10× wash solution. The plugs were transferred into 300 µL of restriction enzyme buffer and incubated for 30 min at 37°C, the buffer was replaced, and 40 U of PmeI was added. Chromosomal DNA was digested overnight and the fragments separated on a 1.2% agarose gel in 0.5× TBE using a CHEF Mapper PFGE system (Bio-Rad) running with a gradient voltage of 6 V/cm, an included angle of 120°, and initial and final switch times of 0.64 sec and 1 min 13.22 sec, respectively, with a run time of 20 h 46 min at 14°C.

### Recombination Assay

Plasmids pTA274 and pTA329 were cut to completion with KpnI or AarI, and purified by excision from agarose gels and extraction using QIAquick columns (Qiagen). Equal aliquots were treated either with shrimp alkaline phosphatase (Promega) or mock-dephosphorylated (no phosphatase). DNA concentration was determined by A_260_, and 0.1–1 µg was used to transform WT, *rad50*, *mre11* and *mre11 rad50* strains. Uncut plasmid was used to measure transformation efficiency. Cells were plated on Hv-Ca (+tryptophan for H292–295 transformed with pTA329). After 5 days incubation, plates were sprayed with Xgal solution and transformants scored the next day. To measure loss of the *trpA* marker in pTA329, red transformants of H292–295 were patched onto Hv-Ca agar without tryptophan, and scored after 3 days incubation.

## Supporting Information

Figure S1 Multiple alignments of Mre11/SbcD and Rad50/SbcC sequences. (A) N-termini of Mre11 and SbcD from *H. volcanii* (Hvo), *Pyrococcus furiosus* (Pfu), *Sulfolobus solfataricus* (Sso), *Bacillus subtilis* (Bsu), *Deinococcus radiodurans* (Dra), *E. coli* (Eco), *Arabidopsis thaliana* (Ath), *Homo sapiens* (Hsa) and *Saccharomyces cerevisiae* (Sce) were aligned using ClustalW. Conserved phosphodiesterase motifs are indicated by I–VI [32]. (B) The conserved regions of Rad50 and SbcC polypeptides were aligned as described above. The conserved motifs in the N-termini are the Walker A (P-loop) and Q-loop, and in the C-termini are the signature motif, Walker B, D-loop and H-loop [32]. The CxxC motif is separated from the N- and C-termini by poorly-conserved coiled-coil regions.
(0.08 MB DOC)

Figure S2 DNA degradation is not affected in *mre11* mutants. (A) Assay for DSB degradation. Plasmid pTA329 is derived from pTA274 and contains a 965 bp *trpA* marker (Trp^+^) inserted after *bgaHa*. If DNA degradation is limited, repair by HR results in a Trp^+^ colony. If degradation extends past *trpA*, HR with *bgaHa-Kp* is possible using downstream sequences (light blue) but the resulting colony will be trp^−^. pTA329 was also cut with AarI, to generate a DSB closer to the *trpA* marker than the KpnI site (472 bp versus 867 bp, respectively). (B) Degradation of cut plasmid is similar in WT and mutant strains. Δ*trpA* derivatives of WT, *rad50*, *mre11* and *mre11 rad50* strains (H292, H293, H294 and H295, respectively) were transformed with pTA329 and plated on Hv-Ca+Trp. Red transformants were replicated onto Hv-Ca to assay the fraction of trp^−^ cells. The efficiency of DSB repair was similar to that observed with pTA274 (data not shown). (C) Assay for single-stranded DNA. The fraction of single-stranded DNA in cells irradiated with UV was measured by slot blotting and hybridization, using denatured DNA as a standard. (D) DNA degradation after UV irradiation is not affected in *mre11/rad50* mutants, as determined by the fraction of single-stranded DNA in WT and *mre11 rad50* cells (H115 and H204, respectively). (E) Deletion end-points of inaccurate end-joining. Plasmid DNA from red WT transformants that failed to cut with StuI was sequenced. Deletions ranged from 6–977 bp and featured end-points with microhomology of 3–5 bp, as indicated. Plasmids that cut with KpnI or StuI were also sequenced, they were identical to the *bgaHa* and *bgaHa-Kp* alleles, respectively (data not shown).
(3.05 MB TIF)

Figure S3 Sequence of *bgaHa* gene used in recombination assays. Nucleotide sequences of the beta-galactosidase genes *bgaH* from *Haloferax alicantei* [58], *bgaHv* from *H. volcanii*, and the hybrid *bgaHa* allele used in this study were aligned. *bgaHa* was constructed by replacement of the native (non-functional) *bgaHv* gene with *bgaH* sequences, between the crossover points shown. Differences between *bgaHa* and the other sequences are indicated by shading. Restriction endonuclease sites used in this study are underlined.
(0.06 MB DOC)

Figure S4 Deletion of *radA* by using plasmid-based complementation. (A) pTA324 is a *pyrE2*-marked (Ura^+^) plasmid carrying a Δ*radA::trpA* construct and integrates at the *radA* locus. pTA411 is a shuttle vector marked with *pyrE2* and *hdrB* (Thy^+^). pTA411 carries the wild-type *radA* gene and facilitates loss of integrated pTA324 by HR. Selection for tryptophan (Trp^+^) ensures that Δ*radA::trpA* cells predominate. Cells are plated on 5-fluoroorotic acid (5-FOA) agar to select for ura^−^ cells, thereby ensuring loss of both integrated and episomal *pyrE2*-marked plasmids. (B) WT, *rad50*, *mre11* and *mre11 rad50* strains (H195, H273, H276, and H280, respectively) were transformed with pTA324 and pTA411. Loss of both plasmids yields 5-FOA-resistant cells (5-FOA^R^, top row), and results in either Δ*radA::trpA* ^+^ or reversion to WT. Almost all colonies obtained in the WT were small, as expected from Δ*radA*. Fewer colonies were obtained in *mre11 rad50* strains and most were large. Small 5-FOA^R^ colonies were patched on complete agar (middle row); two large 5-FOA^R^ colonies, as well as *radA* ^+^ (H195) and Δ*radA* (H112) strains, were included. Cells were transferred to membranes and probed with *radA* sequences (lower row). 88.5% of 5-FOA^R^ Trp^+^ colonies in the WT background were Δ*radA*. In *mre11 rad50* strains, fewer 5-FOA^R^ Trp^+^ colonies proved to be Δ*radA* (3.5%–25%). All *radA* deletions were confirmed by Southern blot (data not shown).
(9.98 MB TIF)

Table S1 Plasmids.
(0.06 MB DOC)

Table S2 Oligonucleotides.
(0.07 MB DOC)

Text S1 Supplemental materials and methods.
(0.05 MB DOC)
