# Supplementary material for: Mre11-Rad50 Promotes Rapid Repair of DNA Damage in the Polyploid Archaeon Haloferax volcanii by Restraining Homologous Recombination
Source: PLoS Genet. 2009 Jul 10;5(7):e1000552. doi: 10.1371/journal.pgen.1000552 (PMC2700283; doi:10.1371/journal.pgen.1000552)
Supplement: Figure S1 — Multiple alignments of Mre11/SbcD and Rad50/SbcC sequences. (A) N-termini of Mre11 and SbcD from H. volcanii (Hvo), Pyrococcus furiosus (Pfu), Sulfolobus solfataricus (Sso), Bacillus subtilis (Bsu), Deinococcus radiodurans (Dra), E. coli (Eco), Arabidopsis thaliana (Ath), Homo sapiens (Hsa) and Saccharomyces cerevisiae (Sce) were aligned using ClustalW. Conserved phosphodiesterase motifs are indicated by I–VI [32]. (B) The conserved regions of Rad50 and SbcC polypeptides were aligned as described above. The conserved motifs in the N-termini are the Walker A (P-loop) and Q-loop, and in the C-termini are the signature motif, Walker B, D-loop and H-loop [32]. The CxxC motif is separated from the N- and C-termini by poorly-conserved coiled-coil regions. (0.08 MB DOC) [file pgen.1000552.s001.doc]

**A**

**Motif I Motif II**

**Hvo MTRVIHTGDTHLGYQQYHSPERRQDFLDAFERVVADALDGDVDAVVHAGDLYHDRRPELPDLLGTLAALRRLDDAG- 76**

Pfu MKFAHLADIHLGYEQFHKPQREEEFAEAFKNALEIAVQENVDFILIAGDLFHSSRPSPGTLKKAIALLQIPKEHS- 75

Sso MVQILHISDTHLGKRQYSLVEREKDIYDIFSQLVDIAIKEHVDVIIHSGDLFDVSSPTTNALVMAIKILKRLKDVN- 76

Bsu MRILHTADWHLGKTLEG-RSRLSEQADVLDELNTIVKDEQIDAIVMAGDAFDTVNPPALAEQLFYESLSALSDRGK 75

Dra MRVLHTADFHAGRLLKG-FDRTPEIHDALVEIAGLARTERADAVLVSGDLFDTGNPSADAEAAVFDFFLRLRDAG- 74

Eco MRILHTSDWHLGQNFYS-KSREAEHQAFLDWLLETAQTHQVDAIIVAGDVFDTGSPPSYARTLYNRFVVNLQQTG- 74

Ath MSREDFSDTLRVLVATDCHLGYMEKD-EIRRHDSFKAFEEICSIAEEKQVDFLLLGGDLFHENKPSRTTLVKAIEILRRHCLNDK 84

Hsa MSTADALDDENTFKILVATDIHLGFMEKD-AVRGNDTFVTLDEILRLAQENEVDFILLGGDLFHENKPSRKTLHTCLELLRKYCMGDR 87

Sce MDYPDPD-TIRILITTDNHVGYNEND-PITGDDSWKTFHEVMMLAKNNNVDMVVQSGDLFHVNKPSKKSLYQVLKTLRLCCMGDK 83

**Motif III**

**Hvo --------------------------------IPFLAIVGNHESTRGG---QWLDLFERLGLATRLGRDPH--------------VVG 115**

Pfu --------------------------------IPVFAIEGNHDRTQRGPSVLNLLEDFGLVYVIGMRKEK----------VENEYLTS 121

Sso --------------------------------IPFLSIPGDHDTPKRKGYLIPHNILSELDLIKILNYEKP-------------YIIK 119

Bsu --------------------------------RPIVVIAGNHDNPDRLSAASPLTHENGIHLIGYPTTEPIH-----IEVPSAGELLA 126

Dra --------------------------------IPGVVIAGNHDSAARLDSVAGLLGWVGIQVVAQPSGDPLAMVREVATK-SGERLRV 129

Eco --------------------------------CHLVVLAGNHDSVATLNESRDIMAFLNTTVVASAGHAPQ-------ILPRRDGTPG 123

Ath PVQFQVVSDQTVNFQN-AFGQVNYEDPHFNVGLPVFSIHGNHDDPAGVDNLSAIDILSACNLVNYFGKMVLGGSGVGQITLYPILMKK 171

Hsa PQVFEILSDQSVNFGFSKFPWVNYQDGNLNISIPVFSIHGNHDDPTGADALCALDILSCAGFVNHFGRSMS----VEKIDISPVLLQK 171

Sce PCELELLSDPSQVFHYDEFTNVNYEDPNFNISIPVFGISGNHDDASGDSLLCPMDILHATGLINHFGKVIE----SDKIKVVPLLFQK 167

**Motif IV**

**Hvo G---VAFYG-----LDHVPRSRRDELD----YQFDPVDADR---------------AVLVAHGLF--------TPFAHADWET-ETVL 167**

Pfu ERLGNGEYL-------VKGVYKDLEIHGMKYMSSAWFEANK---EILKRLFRPTDNAILMLHQGV--------REVSEARGEDYFEIG 191

Sso G---IEVYG-------IPHIPTVSKSILVSALSALRPKSSR---------------SILLLHQGV--------KQILPYDGS--WQME 172

Bsu VG--ALAYPSEARLNEVLSDTF-DEKLLR----DHYDVKIRQAFEHMTSRFRTDAVKIAASHIYVAGGNQ--TDSDGPIEVGGAYTVA 205

Dra G---ALPYLSERRLVKAVDVLGGDLGAQRQKYRENMGFFLRELGRGFEP----GAVNMLMAHTTMDGAVPSGSERTFQLDLTNAYTVS 210

Eco AVLCPIPFLRPRDIITSQAGLNGIEKQQHLLAAITDYYQQHYADACKLRGDQPLPII-ATGHLTTVGASKS--DAVRDIYIGTLDAFP 208

Ath GSTTVALYG--------LGNIR-DERLNRMFQTPHAVQWMRPEVQEG--CDVSDWFNILVLHQ----------NRVKSNPKN---AIS 235

Hsa GSTKIALYG--------LGSIP-DERLYRMFVN-KKVTMLRPKEDEN------SWFNLFVIHQ----------NRSKHGSTN---FIP 230

Sce GSTKLALYG--------LAAVR-DERLFRTFKD-GGVTFEVPTMREG------EWFNLMCVHQ----------NHTGHTNTA---FLP 226

**Motif V Motif VI**

**Hvo AESNVD-FDAVLLGDNHVPDT-----------AELDGTWVTYCGS-TE----RASASERDPRGYN----------LVEFTPDAVDI**-- **226**

Pfu LGDLPEGYLYYALGHIHKRYE-----------TSYSGSPVVYPGS-LE----RWDFGDYEVRYEW----------DIKFKERYGVN-- 251

Sso LGSLPKGFGYYALGHIHTRWR----------LTQDDGSVIAIAGS-PDIM--REEEIGYEKFGKGAY--------LIDFSKDLPIL-- 237

Bsu AESLPADAAYVALGHLHRPQTIKRART----LARYSGSPLAYSFS--EAGYAKSVTIVDAKPGEEATWQEVLLSSGKP--LVKWKA-- 283

Dra GLQLPPGAQYVALGHIHKPQTVSDAP-----LACYPGSVIQLDF--GEAGEKKQINLIEVEAGRPARVEGIPLASGRD---LRTVY-- 286

Eco AQNFPP-ADYIALGHIHRAQIIGGMEH-----VRYCGSPIPLSF--DECGKSKYVHLVTFSNGKLESVENLNVPVTQPMAVLKGDL-- 286

Ath EHFLPRFLDFIVWGHEHECLIDPQEVSGMGFHITQPGSSVATSLIDGES-KPKHVLLLEIK-GNQYRPTKIPLTSVRPFEYTEIVL-- 319

Hsa EQFLDDFIDLVIWGHEHECKIAPTKNEQQLFYISQPGSSVVTSLSPGEA-VKKHVGLLRIK-GRKMNMHKIPLHTVRQFFMEDIVL-- 314

Sce EQFLPDFLDMVIWGHEHECIPNLVHNPIKNFDVLQPGSSVATSLCEAEA-QPKYVFILDIKYGEAPKMTPIPLETIRTFKMKSISL-- 311

**B**

**Walker A (P-loop)**

**Hvo MRFTRIAIRNFKPY-----EDAELDLRDG------VTVIHGVNGSGKSSLLEACFFALYGSK---ALAG--TLEDVVTTGADD--AE 69**

Pfu MKLERVTVKNFRSH-----SDTVVEFKEG------INLIIGQNGSGKSSLLDAILVGLYWP-----LRIKDIKKDEFTKVGAR-DTY 70

Sso MRIDKITLTNFLSH-----EHSEIQFMGE------INVIVGQNGAGKSSIIDGIVFSLFRTHS----RGN--NDNLIRKGSNR--GS 68

Bsu MKPIALSIKGLHSFR----EEQTIDFEGLSG--AGVFGIFGPTGSGKSSILDAMTLALYGK----VERAANNTHGILNHAEDTLSVS 77

Dra MKPLHLTLRGFTAFR----QTTDLDFADL-----ELFALVGPTGSGKSSLLDAMTFALYGET----ARLGATGLDALISQGER-TLS 73

Eco MKILSLRLKNLNSLK----GEWKIDFTREPFASNGLFAITGPTGAGKTTLLDAICLALYHETPR-LSNVSQSQNDLMTRDTAE--CL 80

Ath MSTVDKMLIKGIRSFDPE--NKNVVTFFRP------LTLIVGANGAGKTTIIECLKVSCTGELPPNARSGHSFIHDPKVAGETETKAQ 80

Hsa MSRIEKMSILGVRSFGIEDKDKQIITFFSP------LTILVGPNGAGKTTIIECLKYICTGDFPP-GTKGNTFVHDPKVAQETDVRAQ 81

Sce MSAIYKLSIQGIRSFDSN--DRETIEFGKP------LTLIVGMNGSGKTTIIECLKYATTGDLPP-NSKGGVFIHDPKITGEKDIRAQ 79

**Q-loop**

**Hvo ITLEFVHDGGE-YRIDRRVRVS--GDRATTAKCVL-----DG--P-EGTVEGA---RDVRRHVASLLRMDAEAFVNCAYVQQGEVNKL 143**

Pfu IDLIFEKDGTK-YRITRRFLKGYSSGEIHAMKRLV-----GN--EWKHVTEPS---SKAISAFMEKL-IPYNIFLNAIYIRQGQIDAI 146

Sso VTLYLSNEKDK-IEIIRDIRSTT---EDRLIR--------------NQFPIARSA-TVVSNEIEKILGIDKDIALSTIIVRQGELDKI 137

Bsu FTFALQTNHQISYKVERVFKRTDEMKVKTALCRFIEI-------KDEHTVLADKA-SEVNKRVEELLGLTIDDFTRAVVLPQGKFAEF 157

Dra VALTFEAGGQT-YRVTRTRGRKQADNEVRLDRLDP---------DGEWTGLSSGSQKDIAQRIEDVVGLDFDTFTRCVMLPQGQFAAL 151

Eco AEVGFEVKGEAYRAFWSQNRARNQPDGNLQVPRVELAR------CADGKILADKV-KDKLELTATLTGLDYGRFTRSMLLSQGQFAAF 161

Ath IKLRFKTAAGK-DVVCIRSFQLTQKASKMEYKAIESVLQTINPHTGEKVCLSYRC-ADMDREIPALMGVSKAILENVIFVHQDESNWP 166

Hsa IRLQFRDVNGE-LIAVQRSMVCTQKSKKTEFKTLEGVITRTK--HGEKVSLSSKC-AEIDREMISSLGVSKAVLNNVIFCHQEDSNWP 165

Sce VKLAFTSANGL-NMIVTRNIQLLMKKTTTTFKTLEGQLVAIN-NSGDRSTLSTRS-LELDAQVPLYLGVPKAILEYVIFCHQEDSLWP 164

**Hvo INATPSQRQDMIDDLLQLGKLET----- 166**

Pfu LESDE-AREKVVREVLNLDKFET----- 168

Sso LENFQ-EIMGKILKLELIEKLID----- 159

Bsu LSLKGAERRHMLQRLFNLEQYGD----- 180

Dra LHGKPRQRQELLGELTGMGRVQQ----- 174 **----Coiled-coil----**

Eco LNAKPKERAELLEELTGTEIYGK----- 184

Ath LQDPS-TLKKKFDDIFSATRYTK----- 188

Hsa LSEGK-ALKQKFDEIFSATRYIK----- 187

Sce LSEPS-NLKKKFDEIFQAMKFTK----- 186

**CxxC motif**

**Hvo -----KNARERLAEAERLR-DAGKCPECGQPVEGSP-HVDAISER----- 473**

Pfu  -----GDLKTAIEELKK-A-KGK-CPVCGRELTDE--HREELLSK-----  462

Sso  -----SEKNEIINNISQ-V-EGETCPVCGRPLDEE--HKQKIIKE-----  444

Bsu  -----ARVALLTKELAQKLTAGKPCPVCGSTDHDPSASVHETYEA-----  557

Dra  -----ARMEAGLASYRSHLHEGEPCPLCLQTVHEVP-EGESVDLD-----  486

Eco  **---**--ARIKTLEAQRAQLQ-AGQPCPLCGSTSHPAV-EAYQALEP-----  525

Ath  -----RQMFEPFEKRAR-Q-EHS-CPCCERSFTAD--EEASFIKK-----  713

Hsa  -----AVYSQFITQLTD-E-NQSCCPVCQRVFQTEA-ELQEVISD-----  700

Sce  -----LEFNRKALEIAE-R-DSC-CYLCSRKFENES-FKSKLLQE-----  706

**Hvo -----RNVEVLERMLNETFDLVYAND------------------------------- 755**

Pfu **-----**AALSKIGELASEIFAEFTEG-------------------------------- 762

Sso **-----**-----IENNLNDIISKFDLS-------------------------------- 737

Bsu **-----**LAEEQLESVARDASARLSMLTR------------------------------ 1002

Dra **----Coiled-coil----** **-----**LAEVEAQLLTRAGLLLFDIS-------------------------------- 786

Eco **-----**LTLDNLVHLANQQLTRLHGR-------------------------------- 920

Ath **-----**LIQLKTTEMANKDLDRYYNALDKALMRFHTMKMEEINKIIRELWQQTYRGQD 1174

Hsa **-----**MIVMRTTELVNKDLDIYYKTLDQAIMKFHSMKMEEINKIIRDLWRSTYRGQD 1159

Sce **-----**WVELQTRSFVTDDIDVYSKALDSAIMKYHGLKMQDINRIIDELWKRTYSGTD 1165

**Signature motif Walker B D-loop**

**Hvo -AYSRIRLDGEYG-----------LTVFQKDGTALEPEQ--LSGGERALFNLSLRCAIYRLLAEGIDGAAPLPPLILDEPT-VFLDGG 828**

Pfu -KYSEVVVRAEENKVR--------LFVVWE-GKERPLTF--LSGGERIALGLAFRLAMSLYLAGEIS------LLILDEPT-PYLDEE 831

Sso IKNVEMEIMPKTGRGRS---SSGDILVYTNSGDTLPIVS--LSGGERIALSIALRLAIAKALMSNTN------FFILDEPT-IHLDDQ 813

Bsu -QRYAIEVDSEGG-----------FVMRDDANGGVRRPVSSLSGGETFLTSLSLALALSAQIQLRGEYP--LQFFFLDEGF-GTLDQD 1075

Dra --DGRYRLSLDKGE----------YVVQDLWNAGEVRAVKTLSGGETFLASLSLAIALSDYLAGNKVLG----ALFLDEGF-GTLDPQ 857

Eco -YLLQRKASEALE-----------VEVVDTWQADAVRDTRTLSGGESFLVSLALALALSDLVSHKTR----IDSLFLDEGF-GTLDSE 991

Ath MDYIRIHSDSEGAGTR-----SYSYKVLMQTGDTELEMRGRCSAGQKVLASLIIRLALAETFCLNCG------ILALDEPT-TNLDGP 1250

Hsa IEYIEIRSDADENVSASDKRRNYNYRVVMLKGDTALDMRGRCSAGQKVLASLIIRLALAETFCLNCG------IIALDEPT-TNLDRE 1240

Sce IDTIKIRSDEVSSTVKG---KSYNYRVVMYKQDVELDMRGRCSAGQKVLASIIIRLALSETFGANCG------VIALDEPT-TNLDEE 1243

**H-loop**

**Hvo ----HVSRLVDLVEDMQSRGVKQILIVSHDEDLVGAADDLVRVE-----KNPTTNRSTVERTDAPIVEGALADD 893**

Pfu ----RRRKLITIMERYLKK-IPQVILVSHDEELKDAADHVIRIS-----LENGS--SKVEVVS 882

Sso ----RKAYLIEIIRAAKES-VPQIIVVTHDEEVVQAADYVIRVE-----KRGNK--SFVREET 864

Bsu ----LLDTVVTALEKLQSD-NLAVGVISHVQELRARLPKKLIVHP----AEPSGRGTRVSLELM 1130

Dra ----ALEAVATALENLRTQG-RMVGIVTHVESLSERLPSRLLVS-----KSMAG--SNVIRVDG 909

Eco ----TLDTALDALDALNAS-GKTIGVISHVEAMKERIPVQIKVK-----KINGLGYSKLESAFAMK 1047

Ath NSESLAGALLRIMEDRKGQENFQLIVITHDERFAQMIGQRQHAEKYYRVAKDDMQHSIIEAQEIFD 1316

Hsa NIESLAHALVEIIKSRSQQRNFQLLVITHDEDFVELLGRSEYVEKFYRIKKNIDQCSEIVKCSVSSLGFNVH 1312

Sce NIESLAKSLHNIINMRRHQKNFQLIVITHDEKFLGHMNAAAFTDHFFKVKRDDRQKSQIEWVDINRVTY 1312
