# Supplementary figures and images for: Mre11-Rad50 Promotes Rapid Repair of DNA Damage in the Polyploid Archaeon Haloferax volcanii by Restraining Homologous Recombination
Source: PLoS Genet. 2009 Jul 10;5(7):e1000552. doi: 10.1371/journal.pgen.1000552 (PMC2700283; doi:10.1371/journal.pgen.1000552)

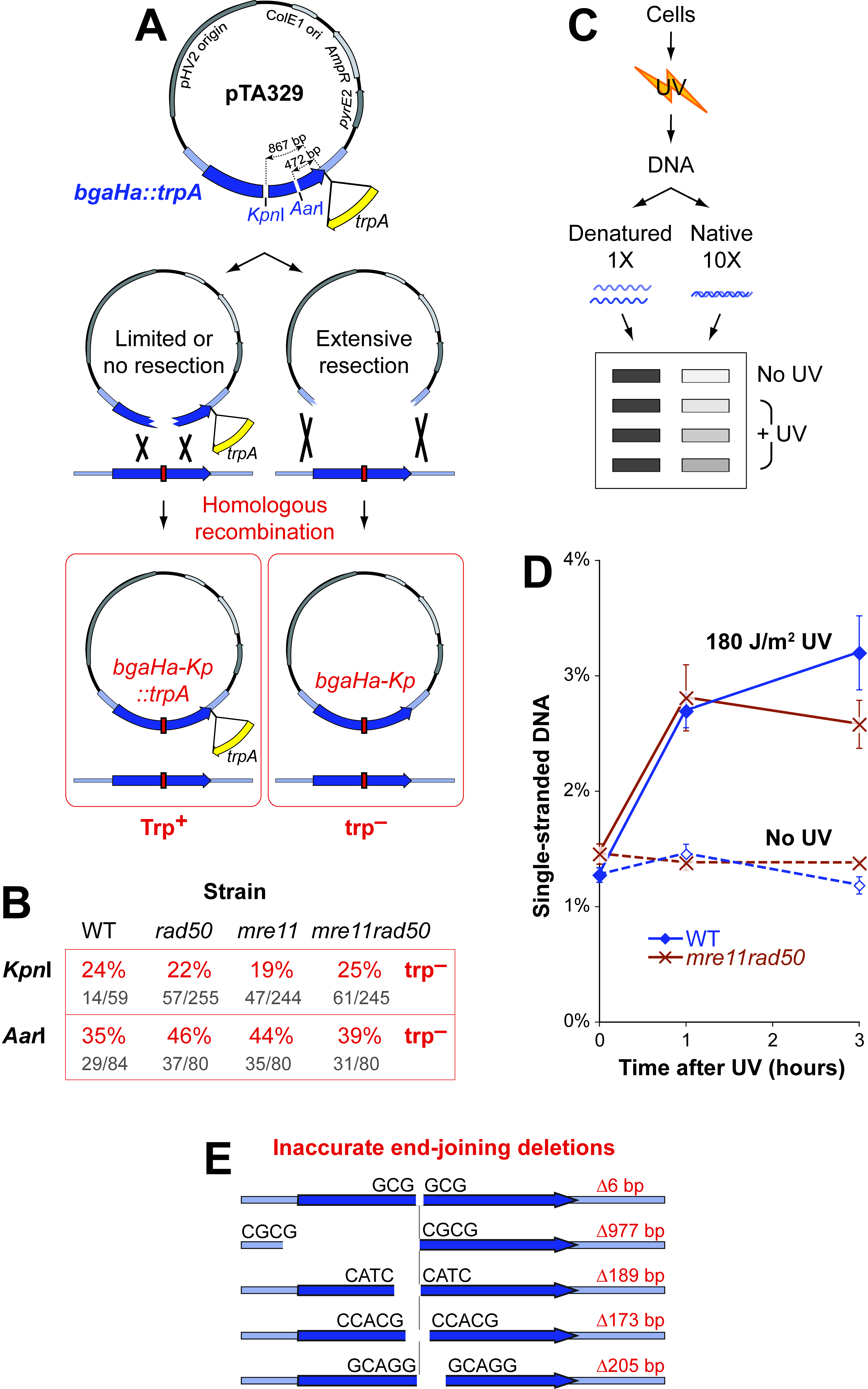

Supplement: Figure S2 — DNA degradation is not affected in mre11 mutants. (A) Assay for DSB degradation. Plasmid pTA329 is derived from pTA274 and contains a 965 bp trpA marker (Trp+) inserted after bgaHa. If DNA degradation is limited, repair by HR results in a Trp+ colony. If degradation extends past trpA, HR with bgaHa-Kp is possible using downstream sequences (light blue) but the resulting colony will be trp−. pTA329 was also cut with AarI, to generate a DSB closer to the trpA marker than the KpnI site (472 bp versus 867 bp, respectively). (B) Degradation of cut plasmid is similar in WT and mutant strains. ΔtrpA derivatives of WT, rad50, mre11 and mre11 rad50 strains (H292, H293, H294 and H295, respectively) were transformed with pTA329 and plated on Hv-Ca+Trp. Red transformants were replicated onto Hv-Ca to assay the fraction of trp− cells. The efficiency of DSB repair was similar to that observed with pTA274 (data not shown). (C) Assay for single-stranded DNA. The fraction of single-stranded DNA in cells irradiated with UV was measured by slot blotting and hybridization, using denatured DNA as a standard. (D) DNA degradation after UV irradiation is not affected in mre11/rad50 mutants, as determined by the fraction of single-stranded DNA in WT and mre11 rad50 cells (H115 and H204, respectively). (E) Deletion end-points of inaccurate end-joining. Plasmid DNA from red WT transformants that failed to cut with StuI was sequenced. Deletions ranged from 6–977 bp and featured end-points with microhomology of 3–5 bp, as indicated. Plasmids that cut with KpnI or StuI were also sequenced, they were identical to the bgaHa and bgaHa-Kp alleles, respectively (data not shown). (3.05 MB TIF) [file pgen.1000552.s002.tif]

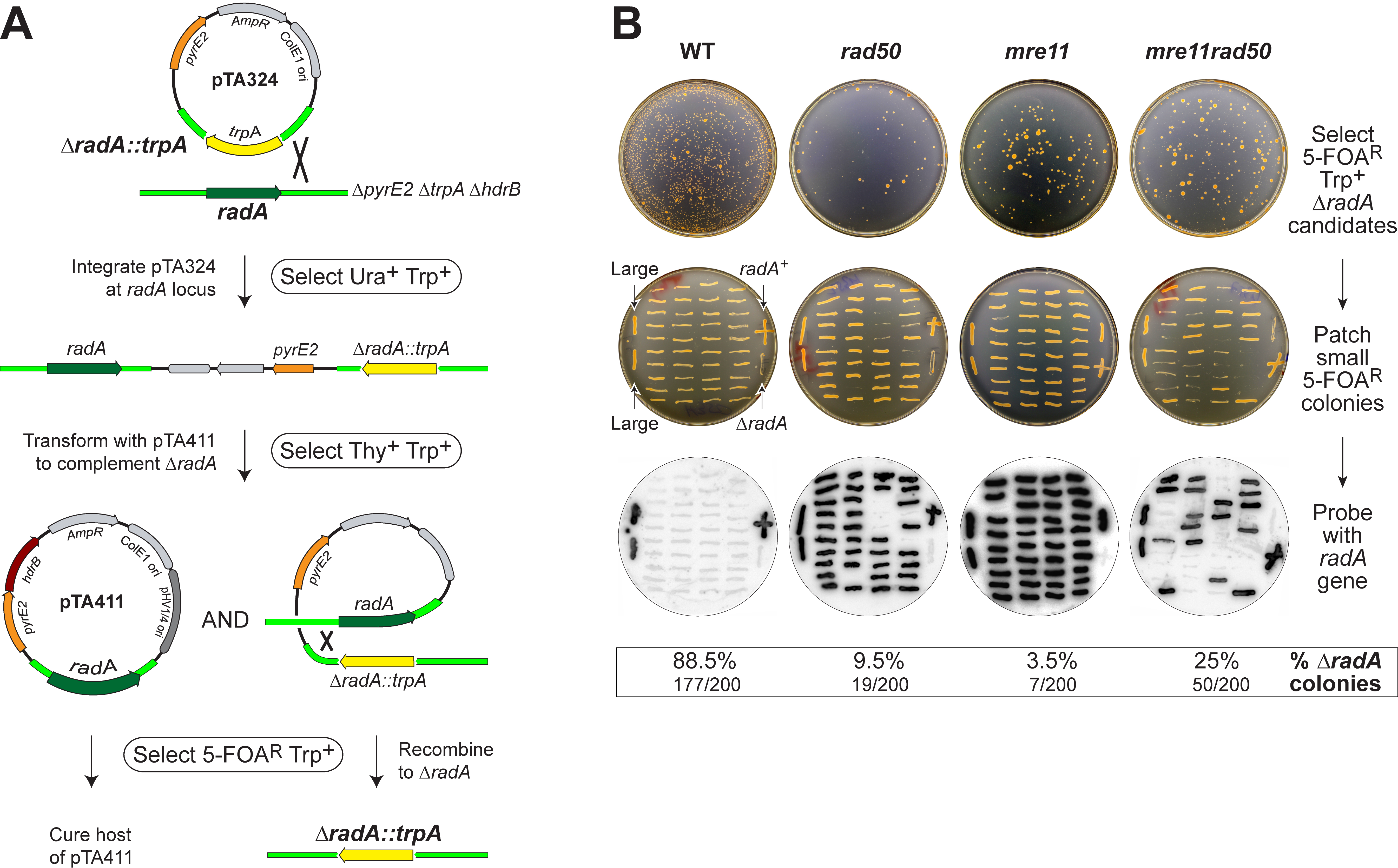

Supplement: Figure S4 — Deletion of radA by using plasmid-based complementation. (A) pTA324 is a pyrE2-marked (Ura+) plasmid carrying a ΔradA::trpA construct and integrates at the radA locus. pTA411 is a shuttle vector marked with pyrE2 and hdrB (Thy+). pTA411 carries the wild-type radA gene and facilitates loss of integrated pTA324 by HR. Selection for tryptophan (Trp+) ensures that ΔradA::trpA cells predominate. Cells are plated on 5-fluoroorotic acid (5-FOA) agar to select for ura− cells, thereby ensuring loss of both integrated and episomal pyrE2-marked plasmids. (B) WT, rad50, mre11 and mre11 rad50 strains (H195, H273, H276, and H280, respectively) were transformed with pTA324 and pTA411. Loss of both plasmids yields 5-FOA-resistant cells (5-FOAR, top row), and results in either ΔradA::trpA + or reversion to WT. Almost all colonies obtained in the WT were small, as expected from ΔradA. Fewer colonies were obtained in mre11 rad50 strains and most were large. Small 5-FOAR colonies were patched on complete agar (middle row); two large 5-FOAR colonies, as well as radA + (H195) and ΔradA (H112) strains, were included. Cells were transferred to membranes and probed with radA sequences (lower row). 88.5% of 5-FOAR Trp+ colonies in the WT background were ΔradA. In mre11 rad50 strains, fewer 5-FOAR Trp+ colonies proved to be ΔradA (3.5%–25%). All radA deletions were confirmed by Southern blot (data not shown). (9.98 MB TIF) [file pgen.1000552.s004.tif]
