# Supplementary material for: Mre11-Rad50 Promotes Rapid Repair of DNA Damage in the Polyploid Archaeon Haloferax volcanii by Restraining Homologous Recombination
Source: PLoS Genet. 2009 Jul 10;5(7):e1000552. doi: 10.1371/journal.pgen.1000552 (PMC2700283; doi:10.1371/journal.pgen.1000552)
Supplement: Figure S3 — Sequence of bgaHa gene used in recombination assays. Nucleotide sequences of the beta-galactosidase genes bgaH from Haloferax alicantei [58], bgaHv from H. volcanii, and the hybrid bgaHa allele used in this study were aligned. bgaHa was constructed by replacement of the native (non-functional) bgaHv gene with bgaH sequences, between the crossover points shown. Differences between bgaHa and the other sequences are indicated by shading. Restriction endonuclease sites used in this study are underlined. (0.06 MB DOC) [file pgen.1000552.s003.doc]

HindIII

*bgaH*  AAGCTTCGAGAGCCACGCGGCGGAGCAGCCGAGACGGGAGGCGGCGACCGCGACGTTGCTTTCCGCACCGGCGGTCCGGA 80

*bgaHa* AAGCTTCGAGAGCCACGCGGCGGAGCAGCCGAGACGGGAGGCGGCGACCGCGACGTTGCTTTCCGCACCGGCGGTCCGGA 80

*bgaHv* AAGCTTCGAGAGCCACGCGGCGGAGCAGCCGAGACGGGAGGCGGCGACCGCGACGTTGCTTTCCGCACCGGCGGTCCGGA 80

*bgaH*  ATTCGAGGGACCGGGCGGTCTCGATTCGCTCGCCCGCCGGCGGGCTCAGCCGAATCATCGTCTCGCCGAACGTGACGAGT 160

*bgaHa* ATTCGAGGGACCGGGCGGTCTCGATTCGCTCGCCCTCCGGCGGGCTCAGCCGAATCATCGTCTCGCCGAACGTGACGAGT 160

*bgaHv* ATTCGAGGGACCGGGCGGTCTCGATTCGCTCGCCCTCCGGCGGGCTCAGCCGAATCATCGTCTCGCCGAACGTGACGAGT 160

*bgaH*  TCAGCCGTCATCGACGCCCCTCGGCGGCTTGCGACCGGGTCTCGCGTTCGATAGCGGAGGCGTTCACGCCGACAGTTAAG 240

*bgaHa* TCAGCCGTCATCGACGCCCCTCGGCGGCTTGCGACCGGGTCTCGCGTTCGATAGCGGAGGCGTTCACACCGACAGTTAAG 240

*bgaHv* TCAGCCGTCATCGACGCCCCTCGGCGGCTTGCGACCGGGTCTCGCGTTCGATAGCGGAGGCGTTCACACCGACAGTTAAG 240

*bgaH*  CCGTTCATGTGTTCGCACGACTGTCTCTCACGGTGGTACATAACTGCGGGCGGTGGGGCCGAAATTTGTGACGGCACCCC 320

*bgaHa* CCGTTCATGTGTTCGCACGACTGTCTCTCACGGTGGTACATAACTGCGGGCGGTGGGGCCGAAATTTGTGACGGCACCCC 320

*bgaHv* CCGTTCATGTGTTCGCACGACTGTCTCTCACGGTGGTACATAACTGCGGGCGGTGGGGCCGAAATTTGTGACGGCACCCC 320

*bgaH*  CGTACGGGAGGACCCGAGTAGTGGATATCAATCGGTGCTCAGACACCGGAAAGAACTATATCTCACCACGTTGATCATTG 400

*bgaHa* CGTACGGGAGGACCCGAGTAGTGGATATCAATCGGTGCTCATACACCGGAAAGAACTATATCTCACCACGTTGATCATTG 400

*bgaHv* CGTACGGGAGGACCCGAGTAGTGGATATCAATCGGTGCTCATACACCGGAAAGAACTATATCTCACCACGTTGATCATTG 400

**Beta-galactosidase start**

*bgaH*  TGTATGACAGTTGGTGTCTGCTATTTCCCGGAGCACTGGTCGCGAGAGCGCTGGGAGACCGATATCAGTCAGATGGCCGA 480

*bgaHa* TGT**ATGACAGTTGGTGTCTGCTATTTCCCGGAGCACTGGTCGCGAGAGCGCTGGGAGACCGATATCAGTCAGATGGCCGA** 480

*bgaHv* TGTATGACAGTTGGTGTCTGCTATTTCCCGGAGCACTGGTCGCGAGAGCGCTGGGAGACCGATATCAGTCAGATGGCCGA 480

BstBI

*bgaH*  GGCTGGAATCGAATACGTTCGAATGGGGGAGTTCGCGTGGCGACGAATCGAACCGGAGCGAGGGACGTTCGATTTCGCGT 560

*bgaHa* **GGCTGGAATCGAATACGTTCGAATGGGGGAGTTCGCGTGGCGACGAATCGAACCGGAGCGAGGGACGTTCGATTTCGCGT** 560

*bgaHv* GGCTGGAATCGAATACGTTCGAATGGGGGAGTTCGCGTGGCGACGAATCGAACCGGAGCGAGGGACGTTCGATTTCGCGT 560

*bgaH*  GGTTAGACGAGGCCGTCGAACTCATCGGGAAGTTCGGTATGAAAGCGGTTCTGTGCACGCCGACCGCGACGCCGCCGAAA 640

*bgaHa* **GGTTAGACGAGACCGTCGAACTCATCGGGAAGTCCGGTATGAAAGCGGTTCTGTGCACGCCGACAGCGACGCCGCCGAAA** 640

*bgaHv* GGTTAGACGAGACCGTCGAACTCATCGGGAAGTCCGGTATGAAAGCGGTTCTGTGCACGCCGACAGCGACGCCGCCGAAA 640

*bgaH*  TGGCTCGTCGACGAACATCCCGACGTTCGACAGCGAGAGCAAGACGGTACGCCGCGTGAGTGGGGGAGCCGTCGGTTCAC 720

*bgaHa* **TGGCTCGTCGACGAACATCCCGACGTTCGACAGCGAGAGCAAGACGGTACGCCGCGTGAGTGGGGGAGCCGTCGGTTCAC** 720

*bgaHv* TGGCTCGTCGACGAACATCCCGACGTTCGACAGCGAGAGCAAGACGGTACGCCGCGTGAGTGGGGGAGCCGTCGGTTCAC 720

*bgaH*  CTGTTTCAACTCACCCACCTACCGTTCCGAGACCGAACGCATCGTTAGCGTGCTGACCGACCGATACGCCGACAACCCCC 800

*bgaHa* **CTGTTTCAACTCACCCACCTACCGTTCCGAGACCGAACGCATCGTTAGCGTGCTGACCGACCGATACGCCGACAACCCCC** 800

*bgaHv* CTGTTTCAACTCACCCACCTACCGTTCCGAGACCGAACGCATCGTTAGCGTGCTGACCGACCGATACGCCGACAACCCCC 800

*bgaH*  ACGTCGCCGGGTGGCAGACTGACAACGAATTCGGCTGTCACGAGACGGTTACCTGCTACTGCGAGGACTGTGGCGAGGCA 880

*bgaHa* **ACGTCGCCGGGTGGCAGACTGACAACGAATTCGGCTGTCACGAGACGGTTACCTGCTACTGCGAGGACTGTGGCGAGGCA** 880

*bgaHv* ACGTCGCCGGGTGGCAGACTGACAACGAATTCGGCTGTCACGAGACGGTTACCTGCTACTGCGAGGACTGTGGCGAGGCA 880

*bgaH*  TTTAGCGAATGGCTCGCCGACCGCTATGAGAGCGTTGCCGACCTCAACGACGCGTGGGGAACGACGTTTTGGAGCCAGCA 960

*bgaHa* **TTTAGCGAATGGCTCGCCGACCGCTATGAGAGCGTTGCCGACCTCAACGACGCGTGGGGAACGACGTTTTGGAGCCAGCA** 960

*bgaHv* TTTAGCGAATGGCTCGCCGACCGCTATGAGAGCGTTGCCGACCTCAACGACGCGTGGGGAACGACGTTTTGGAGCCAGCA 960

*bgaH*  GTACGACGATTTCGAGAGCATCGACCCCCCAAAACCGACACCGGCCGCCAACCACCCTTCGCGGGTACTCGCCTACGAAC 1040

*bgaHa* **GTACGACGATTTCGAGAGTATCGACCCCCCAAAACCGACACCGGCCGCCAACCACCCTTCGCGGGTACTCGCCTACGAAC** 1040

*bgaHv* GTACGACGATTTCGAGAGTATCGACCCCCCAAAACCGACACCGGCCGCCAACCACCCTTCGCGGGTACTCGCCTACGAAC 1040

*bgaH*  GGTTTAGTAACGACAGCGTGGCCGAGTACAACCGCCTGCACGCAGCCCTCATCCGCGAAGCAAACGACGAGTGGTTCGTC 1120

*bgaHa* **GGTTTAGTAACGACAGCGTGGCCGAGTACAACCGCCTGCACGCAGCCCTCATCCGCGAAGCAAACGACGAGTGGTTCGTC** 1120

*bgaHv* GGTTTAGTAACGACAGCGTGGCCGAGTACAACCGCCTGCACGCAGCCCTCATCCGCGAAGCAAACGACGAGTGGTTCGTC 1120

*bgaH*  ACGCACAACTTCATGGGTGGTTTTTCACTCGACGCCTTTCGCCTCGCCGCCGACCTCGATTTCCTCTCGTGGGACTCCTA 1200

*bgaHa* **ACGCACAACTTCATGGGTGGTTTTTCACTCGACGCCTTTCGCCTCGCCGACGACCTCGATTTCCTCTCGTGGGACTCCTA** 1200

*bgaHv* ACGCACAACTTCATGGGTGGTTTTTCACTCGACGCCTTTCGCCTCGCCGACGACCTCGATTTCCTCTCGTGGGACTCCTA 1200

*bgaH*  TCCGACGGGGTTCGTGCAGGACCGACAGCCGGACACGCCGACGGTCGACGAATTACGAGCGGGGAACCCCGACCAAGTGA 1280

*bgaHa* **TCCGACGGGGTTCGTGCAGGACCGACAGCCGGACACGCCGACGGTCGACGAATTACGAGCGGGGAACCCCGACCAAGTGA** 1280

*bgaHv* TCCGACGGGGTTCGTGCAGGACCGACAGCCGGACACGCCGACGGTCGACGAATTACGAGCGGGGAACCCCGACCAAGTGA 1280

*bgaH*  GCATGAACCACGACCTCCAACGCGGCGCGAAAGGAAAGCCGTTCTGGGTGATGGAGCAACAGCCGGGAGACATCAACTGG 1360

*bgaHa* **GCATGAACCACGACCTCCAGCGCGGCGCGAAAGGAAAGCCGTTCTGGGTGATGGAGCAACAGCCGGGAGACATCAACTGG** 1360

*bgaHv* GCATGAACCACGACCTCCAGCGCGGCGCGAAAGGAAAGCCGTTCTGGGTGATGGAGCAACAGCCGGGAGACATCAACTGG 1360

*bgaH*  CCGCCGCAGTCGCCGCAACCGGCCGACGGGGCGATGCGCCTGTGGGCTCACCACGCCGTCGCCCACGGCGCGGACGCCGT 1440

*bgaHa* **CCGCCGCAGTCGCCGCAACCGGCCGACGGGGCGATGCGCCTGTGGGCTCACCACGCCGTCGCCCACGGCGCGGACGCCGT** 1440

*bgaHv* CCGCCGCAGTCGCCGCAACCGGCCGACGGGGCGATGCGCCTGTGGGCTCACCACGCCGTCGCCCACGGCGCGGACGCCGT 1440

*bgaH*  CGTCTACTTCCGCTGGCGTCGCTGTCGTCAAGGGCAAGAACAGTACCACGCCGGGCTTCGGCGGCAGGACGGCTCTCCGG 1520

*bgaHa* **CGTCTACTTCCGCTGGCGTCGCTGTCGTCAAGGGCAAGAACAGTACCACGCCGGGCTTCGGCGGCAGGACGGCTCTCCGG** 1520

*bgaHv* CGTCTACTTCCGCTGGCGTCGCTGTCGTCAAGGGCAAGAACAGTACCACGCCGGGCTTCGGCGGCAGGACGGCTCTCCGG 1520

KpnI

*bgaH*  ACCGCGGGTACCGCGAAGCATCGACCGCCGCCGACGAACTGTTCGACTTGGATTCGGTCGATGCGTCAGTCGCGCTCGTC 1600

*bgaHa* **ACCGCGGGTACCGCGAAGCATCGACCGCCGCCGACGAACTGTTCGACTTGGATTCGGTCGATGCGTCAGTCGCGCTCGTC** 1600

*bgaHv* ACCGCGGGTACCGCGAAGCATCGACCGCCGCCGACGAACTGTTCGACTTGGATTCGGTCGATGCGTCAGTCGCGCTCGTC 1600

*bgaH*  CACGACTACGAGAGCCTGTGGGCGACGCGTTCGCAACCTCTCTCGCCCGACTGGGACTACTGGAACCACTTACGGACGTA 1680

*bgaHa* **CACGACTACGAGAGCCTGTGGGCGACGCGTTCGCAACCTCTCTCGCCCGACTGGGACTACTGGAACCACTTACGGACGTA** 1680

*bgaHv* CACGACTACGAGAGCCTGTGGGCGACGCGTTCGCAACCTCTCTCGCCCGACTGGGACTACTGGAACCACTTACGGACGTA 1680

Upstream crossover: *bgaHv~bgaH*

*bgaH*  CTACGATGCGCTTCGCGC-------------------------------------------------------------- 1698

*bgaHa* **CTACGATGCGCTTCGCGC--------------------------------------------------------------** 1698

*bgaHv* CTACGATGCGCTTCGCGCAGACGTTGAGCCTCCCTCCGGAGTAACCTCATCCTTACGGACGTACTACGATGCGCTTCGCG 1760

*bgaH*  -CCGCGGCGTGCAGGTCGACATCGTCTCGCCGGAAGCGACTCTCGAACGGTACGACGCGGTCGTCGCACCGACTTTGTAT 1777

*bgaHa* **-CCGCGGCGTGCAGGTCGACATCGTCTCGCCGGAAGCGACTCTCGAACGGTACGACGCGGTCGTCGCACCGACTTTGTAT** 1777

*bgaHv* CCCGCGGCGTGCAGGTCGACATCGTCTCGCCGGAAGCGACTCTCGAACGGTACGACGCGGTCGTCGCACCGACTTTGTAT 1840

*bgaH*  CTCGTCGGCGACGAACTGTCGACCGCGCTGACCGACTACGTCGATTCGGGTGGCTGTCTCCTCCTCGGTGCTCGAACGGG 1857

*bgaHa* **CTCGTCGGCGACGAACTGTCGACCGCGCTGACCGACTACGTCGATTCGGGTGGCTGTCTCCTCCTCGGTGCTCGAACGGG** 1857

*bgaHv* CTCGTCGGCGACGAACTGTCGACCGCGCTGACCGACTACGTCGATTCGGGTGGCTGTCTCCTCCTCGGTGCTCGAACGGG 1920

AarI

*bgaH*  GGAGAAAGACCCGTACAACCGGCTTCACGAGTCGCTCGCGCCGGGACCACTCACCGCTCTCACCGGCGCGCAGGTGGCGC 1937

*bgaHa* **GGAGAAAGACCCGTACAACCGGCTTCACGAGTCGCTCGCGCCGGGACCACTCACCGCTCTCACCGGCGCGCAGGTGGCGC** 1937

*bgaHv* GGAGAAAGACCCGTACAACCGGCTTCACGAGTCGCTCGCGCCGGGACCACTCACCGCTCTCACCGGCGCGCAGGTGGCGC 2000

*bgaH*  GTCACGAAACGCTTCCCGACCACGTCGAGACGCGACTCTCCTACGACGGTGCGACGTACGAGTTCCGAACGTGGGCTTCG 2017

*bgaHa* **GTCACGAAACGCTTCCCGACCACGTCGAGACGCGACTCTCCTACGACGGTGCGACGTACGAGTTCCGAACGTGGGCTTCG** 2017

*bgaHv* GTCACGAAACGCTTCCCGACCACGTCGAGACGCGACTCTCCTACGACGGTGCGACGTACGAGTTCCGAACGTGGGCTTCG 2080

*bgaH*  TGGCTGGCTCCCGAAGTCGGAGTTCCACGAGGCGAGTATCGGACGGGTGAAGCAGCCGGAAACACCGCAATCGTGCGGAA 2097

*bgaHa* **TGGCTGGCTCCCGAAGTCGGAGTTCCACGAGGCGAGTATCGGACGGGTGAAGCAGCCGGAAACACCGCAATCGTGCGGAA** 2097

*bgaHv* TGGCTGGCCCCCGAAGTCGGAGTTCCACGAGGCGAGTACCGGACGGGTGAAGCAGCCGGAAACACCGCAATCGTGCGGAA 2160

*bgaH*  CGCCGCCGGAGACGGGAGCGTGACGTACTGTGGCTGCTGGCCGGGGGACGACCTTGCCGACGCACTCGTGACAGAGTTAC 2177

*bgaHa* **CGCCGCCGGAGACGGGAGCGTGACGTACTGTGGCTGCTGGCCGGGGGACGACCTTGCCGACGCACTCGTGACAGAGTTAC** 2177

*bgaHv* CGCCGCCGGAGACGGGAGCGTGACGTACTGTGGCTGCTGGCCGGGGGACGACCTTGCCGACGCACTCGTGACAGAGTTAC 2240

*bgaH*  TGGACGCCGCCGGCGTCGAGTACACTGAGCGATTCCCGGACGGCGTGCGCGTGATGGAGCGCGACGGCTATACGTGGGCG 2257

*bgaHa* **TGGACGCCGCCGGCGTCGAGTACACTGAGCGATTCCCGGACGGCGTGCGCGTGATGGAGCGCGACGGCTATACGTGGGCG** 2257

*bgaHv* TGGACGCCGCCGGCGTCGAGTACACTGAGCGATTCCCGGACGGCGTGCGCGTGATGGAGCGCGACGGCTATACGTGGGCG 2320

Downstream crossover: *bgaH~bgaHv*

*bgaH*  CTTAACTTCACGAGCGACCCGGTGACGTTGACCGTCCCCGATTCCACCGGGTTCCTGCTCGGTGAGTCCACCGTCGACGT 2337

*bgaHa* **CTTAACTTCACGAGCGACCCGGTGACGTTGACCGTCCCCGATTCCACCGGGTTCCTGCTCGGTGAGTCCACCGTCG----** 2333

*bgaHv* CTTAACTTCACGAGCGACCCGGTGACGTTGACCGTCCCCGATTCCACCGGGTTCCTGCTCGGTGAGTCCACCGTCG---- 2396

BamHI **Beta-galactosidase end**

*bgaH*  CGACGCGTTCGATACCGCGGTACTCGACGGGTCCATCCGAGGTGTCGGACTCGCGTCCGAGTGAGTCGGCACGGGACCAC 2417

*bgaHa* **--ACGCGTTCGATACCGCGGTACTCGACGGATCCATCCGAGGTGTCGGACTCGCGTCCGAGTGA**GTCGGCACGGGACCAC 2411

*bgaHv* --ACGCGTTCGATACCGCGGTACTCGACGGATCCATCCGAGGTGTCGGACTCGCGTCCGAGTGAGTCGGCACGGGACCAC 2474

*bgaH*  TCGCCAACGCGACTCTCGCCCGGCGTGTTCTCGAACGCCGAAGAGAATAACGGCCGGACCGTGCCCCGATGAGGCACCGA 2497

*bgaHa* TCGCCAACGCGGCTCTCGCCCGGCGTGTTCTCGAACGCCGAAGAGAATAACGGCCGGACCGTGCCCCGATGAGGCACCGA 2491

*bgaHv* TCGCCAACGCGGCTCTCGCCCGGCGTGTTCTCGAACGCCGAAGAGAATAACGGCCGGACCGTGCCCCGATGAGGCACCGA 2554

*bgaH*  CCGGACGCAGGTTCGCCGTCACGGCGCGTTGAACGTCCACTTCTGGTTGTCGCCGCCGACTTCGGTGTACTGGGGTGTTG 2577

*bgaHa* CCGGACGCTGGTTCGCCGTCACGGCGCGTTGAACGTCCACTTCTGGTTGTCGCCGCCGACTTCGGTGTACTGGGGTGTTG 2571

*bgaHv* CCGGACGCTGGTTCGCCGTCACGGCGCGTTGAACGTCCACTTCTGGTTGTCGCCGCCGACTTCGGTGTACTGGGGTGTTG 2634

*bgaH*  ATTCCTCGCGTGTACACACGCGGAAGCGCGTCACAACTGCGTCTGTGGGGCGTTGAGGGTGTCCGTCTTCGTCTTG-GGG 2656

*bgaHa* ATTCCTCGCGTGTACACACGCGGAAGCGCGTCACAACTGCGTCTGTGGGGCGTTGAGGGTGTCCGTCTTCGTCTTGAGGG 2651

*bgaHv* ATTCCTCGCGTGTACACACGCGGAAGCGCGTCACAACTGCGTCTGTGGGGCGTTGAGGGTGTCCGTCTTCGTCTTGAGGG 2714

*bgaH*  CCGTCCCGGACTGCGCGTCGAAGAGGTAGGCCGCCTCGTCTCGGAAGCTAATTCCGACGCGGTCGCCGGTCTCGGGGCGA 2736

*bgaHa* CCGTCCCGGACTGCGCGTCGAAGAGGTAGGCCGCCTCGTCTCGGAAGCTAATTCCGACGCGGTCGCCGGTCTCGGGGCGA 2731

*bgaHv* CCGTCCCGGACTGCGCGTCGAAGAGGTAGGCCGCCTCGTCTCGGAAGCTAATTCCGACGCGGTCGCCGGTCTCGGGGCGA 2794

*bgaH*  ATCGACGCGTCGGTTCGGGCGACAATCTCTTCTTGCTAAACCACGGTGATTTGCTGTTCGACGACAACGACGCGCTCCAC 2816

*bgaHa* ATCGACGCGTCGGTTCGGGCGACAATCTCTTCTTGCTAAACCACGGTGATTTGCTGTTCGACGACAACGACGCGCTCCAC 2811

*bgaHv* ATCGACGCGTCGGTTCGGGCGACAATCTCTTCTTGCTAAACCACGGTGATTTGCTGTTCGACGACAACGACGCGCTCCAC 2874

*bgaH*  GTGTACACCGTCGTCGACGGGCAACTCTACGCCGGGGTTG-CGACAGCATCCTCGGGGTGGACCGA-TGGACGCTCTACT 2894

*bgaHa* GTGTACACCGTCGTCGACGGGCAACTCTACGCCGGGGTTGGCGACAGCATCCTCGGGGTGGACCGACTGGACGCTCTACT 2891

*bgaHv* GTGTACACCGTCGTCGACGGGCAACTCTACGCCGGGGTTGGCGACAGCATCCTCGGGGTGGACCGACTGGACGCTCTACT 2954

*bgaH*  GTCTCCACGAGGGCCCGTTCTGTGGAGTGGACGGTCGAAAACACGATAGATATCGGTGGGAACACGAGAGGGTCCTGTCG 2974

*bgaHa* GTCTCCACGAGGGCCCGTTCTGTGGAGTGGACGGTCGAAAACACGATAGATATCGGTGGGAACACGAGAGGGTCCTGTCG 2971

*bgaHv* GTCTCCACGAGGGCCCGTTCTGTGGAGTGGACGGTCGAAAACACGATAGATATCGGTGGGAACACGAGAGGGTCCTGTCG 3034

ScaI

*bgaH*  ATACCACTCGAACGGCCCCGCGAGAACGGCACGGCGTTCGCACTCCAGGAGTACT 3029

*bgaHa* ATACCACTCGAACGGCCCCGCGAGAACGGCACGGCGTTCGCACTCCAGGAGTACT 3026

*bgaHv* ATACCACTCGAACGGCCCCGCGAGAACGGCACGGCGTTCGCACTCCAGGAGTACT 3089
