# Supplementary material for: Mre11-Rad50 Promotes Rapid Repair of DNA Damage in the Polyploid Archaeon Haloferax volcanii by Restraining Homologous Recombination
Source: PLoS Genet. 2009 Jul 10;5(7):e1000552. doi: 10.1371/journal.pgen.1000552 (PMC2700283; doi:10.1371/journal.pgen.1000552)
Supplement: Table S1 — Plasmids. (0.06 MB DOC) [file pgen.1000552.s005.doc]

**Table S1.** Plasmids.

| **Plasmid** | **Relevant properties** | **Source** |
| --- | --- | --- |
| pGB70 | Integrative vector based on pUC19, with *pyrE2* marker | [35] |
| pTA42 | pBluescript II with *H. volcanii* 5333 bp MluI fragment containing *mre11-rad50* operon | This study |
| pTA80 | pGB70 with 2831 bp KpnI–HindIII fragment from pMDS41 containing *∆radA* construct [41] | This study |
| pTA95 | pGB70 with *∆trpA* construct | [55] |
| pTA102 | pGB70 with 3439 bp HindIII–Sau3A *H. alicantei* genomic DNA fragment from pMLH32 containing β-galactosidase gene *bgaH* [58] | This study |
| pTA128 | pBluescript I with 3310 bp HindIII–AgeI genomic fragment isolated from H54, containing recombinant β-galactosidase gene *bgaHa* | This study |
| pTA131 | Integrative vector based on pBluescript II, with *pyrE2* marker | [55] |
| pTA137 | pTA42 with deletion of 2522 bp BspEI–BsaBI fragment of *rad50* to generate *∆rad50*, insertion of *pyrE2* marker from pGB70 at PsiI site | This study |
| pTA138 | pTA42 with replacement of 2431 bp BsiWI fragment containing *mre11* by 1108 bp PCR fragment containing *∆mre11*, insertion of *pyrE2* marker from pGB70 at PsiI site | This study |
| pTA151 | pTA131 with 3354 bp HindIII–AgeI fragment containing *bgaHa-Bb* allele derived from pTA128 by insertion of 26 bp oligonucleotide bgaBb at the BstBI site in *bgaHa* | This study |
| pTA154 | pTA131 with 3354 bp HindIII–AgeI fragment containing *bgaHa-Kp* allele derived from pTA128 by insertion of 26 bp oligonucleotide bgaKp at the KpnI site in *bgaHa* | This study |
| pTA171 | pTA138 with deletion of 2522 bp BspEI–BsaBI fragment of *rad50*, to generate *∆mre11rad50* | This study |
| pTA230 | *E. coli/H. volcanii* shuttle vector derived from pTA131, with *pyrE2* marker and pHV2 replication origin | [55] |
| pTA274 | pTA230 with 3022 bp HindIII–ScaI fragment of pTA128 containing *bgaHa* and flanking sequence | This study |
| pTA329 | pTA274 with 965 bp *trpA* marker [55] inserted at BamHI site directly after *bgaHa* | This study |
| pTA324 | pTA131 with 2093 bp *∆radA::trpA^+^* construct: KpnI–EcoRI fragment of pSJS1140 [6] with 985 bp NcoI–NotI fragment of *radA* replaced by 965 bp *trpA* marker [55] | This study |
| pTA354 | Shuttle vector based on pBluescript II, with *pyrE2* marker and *ori-pHV1/4* replication origin | [57] |
| pTA409 | Shuttle vector based on pBluescript II, with *pyrE2* and *hdrB* markers and *ori-pHV1/4* replication origin [57] | This study |
| pTA411 | pTA409 with 1634 bp KpnI–BstBI fragment of pSJS1140 [41] containing *radA* gene under its native promoter | This study |
| pTA641 | pTA354 with 3022 bp HindIII–ScaI fragment of pTA128 containing *bgaHa* and flanking sequence, similar to p274 but with *ori-pHV1/4* replication origin for transformation of *radA* mutant H607 | This study |
| pTA795 | pTA354 with 5602 bp PvuII fragment of pTA42 containing *mre11-rad50* operon under its native promoter | This study |
