# Supplementary material for: Mre11-Rad50 Promotes Rapid Repair of DNA Damage in the Polyploid Archaeon Haloferax volcanii by Restraining Homologous Recombination
Source: PLoS Genet. 2009 Jul 10;5(7):e1000552. doi: 10.1371/journal.pgen.1000552 (PMC2700283; doi:10.1371/journal.pgen.1000552)
Supplement: Table S2 — Oligonucleotides. (0.07 MB DOC) [file pgen.1000552.s006.doc]

**Table S2.** Oligonucleotides.

| **Primer** | **Sequence (5'–3')*^a^*** | **Relevant properties** |
| --- | --- | --- |
| HvR5BF | TCTCGACGCTCGAATCGA | *rad50* probe, forward PCR primer |
| HvR5BR | TTCGTCGTCGGGTTCTTCT | *rad50* probe, reverse PCR primer |
| dMre5F | GCATTGCTTGGTGTATCGTACG | *mre11* deletion, upstream forward PCR primer, BsiWI site used to replace *mre11* gene, generating *∆mre11* construct |
| dMre5R | CACCCGTGA**CAT**GTATCACTCGG | *mre11* deletion, upstream reverse PCR primer, PciI site used to ligate with BspHI site in dMre3F (*mre11* start codon, bold) |
| dMre3F | GCTGGGTGATTTCTC**ATG**AGATTCACC | *mre11* deletion, downstream forward PCR primer, BspHI site used to ligate with PciI site in dMre5R (*rad50* start codon, bold) |
| dMre3R | TCGCGTTGATGAGCTTGTTGACCTCG | *mre11* deletion, downstream reverse PCR primer. BsiWI site (16 bp internal to primer) used for *∆mre11* construct (see dMre5F) |
| bgaKpF | ACACCGTG**AGGCCT**CAGCTGCCGTAC | Top strand of double-stranded (ds) oligonucleotide bgaKp, inserted into KpnI site of *bgaHa* (StuI site, bold) |
| bgaKpR | GGCAGCTG**AGGCCT**CACGGTGTGTAC | Bottom strand of ds oligonucleotide bgaKp (see bgaKpF) |
| bgaBbF | CGAGCTCGGCACCATGGCTGAGACGT | Top strand of ds oligonucleotide bgaBb, inserted into BstBI site of *bgaHa* |
| bgaBbR | CGACGTCTCAGCCATGGTGCCGAGCT | Bottom strand of ds oligonucleotide bgaBb (see bgaBbF) |
| bgaH3W | GATCCATCCGAGGTGTCGGACTCGCGTCCGAG**TGA**G | Top strand of ds oligonucleotide bgaH3, inserted into BamHI site on *bgaHa-*proximal side of *trpA* marker, to restore *bgaHa* coding sequence (*bgaHa* stop codon, bold) |
| bgaH3C | GATCC**TCA**CTCGGACGCGAGTCCGACACCTCGGATG | Bottom strand of ds oligonucleotide bgaH3 (see bgaH3W) |

*^a^* Restriction endonuclease sites or cohesive ends used in cloning are underlined
