# Supplementary material for: Mre11-Rad50 Promotes Rapid Repair of DNA Damage in the Polyploid Archaeon Haloferax volcanii by Restraining Homologous Recombination
Source: PLoS Genet. 2009 Jul 10;5(7):e1000552. doi: 10.1371/journal.pgen.1000552 (PMC2700283; doi:10.1371/journal.pgen.1000552)
Supplement: Text S1 — Supplemental materials and methods. (0.05 MB DOC) [file pgen.1000552.s007.doc]

**Supplemental Materials and Methods**

**Isolation of β-galactosidase Gene *bgaHa* and Construction of DSB Repair Assay Plasmid pTA274**

The β-galactosidase gene *bgaHv* of *H. volcanii* is not functional when assayed with Xgal. However, the *bgaH* gene from *Haloferax alicantei* is active in *H. volcanii* and yields colonies that stain blue with Xgal [58]. To replace *bgaHv* with a functional β-galactosidase gene, a 3439 bp HindIII–Sau3A *H. alicantei* DNA fragment from pMLH32 [58] was cloned in the *pyrE2*-marked plasmid pGB70 [35] to generate pTA102. The *∆pyrE2* strain H26 [55] was transformed with pTA102, and a gene knockout/replacement system [35,55] was used to isolate clones that stained blue when sprayed with Xgal. One blue-staining clone was designated H54 and used to generate a genomic library from which the recombinant β-galactosidase gene *bgaHa* was isolated as a 3310 bp HindIII–AgeI fragment. The *bgaHa* fragment was cloned in pBluescript to generate pTA128 and its sequence was compared to the *H. volcanii* *bgaHv* and *H. alicantei* *bgaH* genes (Figure S3). A 3022 bp HindIII–ScaI fragment of pTA128 containing the *bgaHa* gene was subcloned in the shuttle vector pTA230 [55] to generate pTA274.

**Construction of DSB Repair Assay Plasmid pTA329**

A 965 bp fragment containing the *trpA* marker [55] was inserted in pTA274 at a BamHI site located 30 bp before the end of the *bgaHa* coding sequence. To restore *bgaHa* function, the 36 bp double-stranded oligonucleotide bgaH3, comprising the terminal 30 bp of *bgaHa*, was inserted at the BamHI site on the *bgaHa*-proximal side of the *trpA* marker. A plasmid with the correct location and orientation of the bgaH3 oligonucleotide was verified by sequencing, and designated pTA329 (Figure S2A).

**Construction of *bgaHa-Kp* and *bgaHa-Bb* Alleles**

To ensure that plasmid-borne and chromosomal copies of *bgaHa* are identical (other than engineered mutations), all strains used in this study are derived from H54 (apart from H26 and H112). The *bgaHa-Kp* allele was generated by inserting the 26 bp double-stranded oligonucleotide bgaKp at the KpnI site of *bgaHa* in pTA128. A 3310 bp HindIII–AgeI fragment containing *bgaHa-Kp* was subcloned in the *pyrE2* plasmid pTA131 to generate pTA154, and used to replace the chromosomal *bgaHa* gene as described previously [35,55]; the resulting *bgaHa-Kp* strain was designated H115. The *bgaHa-Bb* allele was generated in the same manner, by inserting the 26 bp oligonucleotide bgaBb at the BstBI site in *bgaHa*, and subcloning in pTA131 to generate pTA151. Recombination between this construct and the chromosomal *bgaHa-Kp* allele was used to generate *bgaHa* derivatives of *bgaHa-Kp* strains H115 and H204; the resulting strains were designated H642 and H645, respectively, and stain blue with Xgal.

**Construction of Shuttle Vector pTA409**

A synthetic operon was constructed consisting of *H. volcanii* *pyrE2* and *hdrB* genes under control of the *Halobacterium salinarum* ferredoxin promoter. A 685 bp PCR product of the *hdrB* coding sequence [59], was inserted at the XbaI–HindIII sites downstream of *pyrE2* in pGB70 [35]. To generate pTA409, the 1253 bp *pyrE2::hdrB* operon was inserted at the PsiI site in pBluescript II and a 948 bp BmgBI–AciI fragment containing the *H. volcanii* *ori-pHV1/4* DNA replication origin [57] was inserted at the PciI site.

**Deletion of *radA* by Using Plasmid-based Complementation**

We attempted to delete *radA* by a gene knockout system [35]. The *pyrE2* gene for uracil synthesis is used to select for integration of a gene deletion construct and resistance to 5-fluoroorotic acid (5-FOA^R^) is used to counter-select for loss of the construct by HR. Since there is no bias for gene deletion, it is difficult to generate mutants with a deleterious phenotype if these are outcompeted by WT cells. When deleting *radA* in a WT background, <2% of 5-FOA^R^ colonies proved to be *∆radA* (data not shown). Attempts to delete *radA* by this method in the *mre11 rad50* mutants were unsuccessful. A variant of the knockout system, where *radA* is replaced with a *trpA* marker to select for *∆radA::trpA^+^* mutants [55], was also unsuccessful and yielded only merodiploid cells heterozygous for *radA^+^/∆radA::trpA^+^* (data not shown). Maintenance of heterozygosity is probably due to the requirement for RadA in HR [41], coupled with the high ploidy of *H. volcanii* [4]. Excision of the deletion construct to generate *∆radA::trpA^+^* leads to reduced levels of RadA, attenuating HR and thereby preventing excision of the remaining copies of the deletion construct.

We therefore developed the protocol shown in Figure S4A, where a plasmid-borne *radA* gene complements *radA* deletion and facilitates efficient HR. Counter-selection for 5-FOA^R^ ensures loss of both integrated (pTA324) and episomal (pTA411) *pyrE2*-marked plasmids. *∆pyrE2 ∆trpA ∆hdrB* strains were transformed with pTA324 and integration of pTA324 at the *radA* locus was verified by Southern blot (data not shown). Integrants were transformed with pTA411, then grown for ~30 generations in Hv-Ca broth, ~30 generations in Hv-Ca broth +thymidine +uracil, and plated on Hv-Ca +5-FOA +thymidine. 5-FOA^R^ Trp^+^ cells were transferred to nylon membranes and probed with a 985 bp NcoI–NotI fragment of *radA* from pSJS1140 [41]. This new protocol enabled the deletion of *radA* in both WT and *mre11 rad50* strains, although the isolation of *∆radA* colonies proved much rarer in the latter (Figure S4B).

**Determination of Total ssDNA**

A 200 µl aliquot of cells was centrifuged and lyzed in an equal volume of 10 mM Tris.HCl, 0.1 M EDTA pH8. Genomic DNA was purified by two rounds of chloroform extraction and RNA removed by treatment with RNase A (~33 µg) for 1 hour at 45°C. DNA samples were left overnight at 4°C to resuspend fully. A 10 µl aliquot was used to determine the fraction of single-stranded DNA by using slot blotting as described previously [60]. Membranes were probed with total *H. volcanii* genomic DNA.

**Supplemental References**

58. Holmes ML, Dyall-Smith ML (2000) Sequence and expression of a halobacterial beta-galactosidase gene. Mol Microbiol 36: 114-122.

59. Ortenberg R, Rozenblatt-Rosen O, Mevarech M (2000) The extremely halophilic archaeon *Haloferax volcanii* has two very different dihydrofolate reductases. Mol Microbiol 35: 1493-1505.

60. Garvik B, Carson M, Hartwell L (1995) Single-stranded DNA arising at telomeres in *cdc13* mutants may constitute a specific signal for the *RAD9* checkpoint. Mol Cell Biol 15: 6128-6138.
